# Global discovery of RNA modifications and functional analysis of m^5^C methylome in cyanobacteria

**DOI:** 10.1016/j.jbc.2026.111133

**Published:** 2026-01-07

**Authors:** Gaoxiang Cao, Mingtian Ling, Jiao Zhan, Jian Lin, Li Yuan, Mingkun Yang, Feng Ge

**Affiliations:** 1 Key Laboratory of Breeding Biotechnology and Sustainable Aquaculture, Institute of Hydrobiology, Chinese Academy of Sciences, Wuhan, China; 2 University of Chinese Academy of Sciences, Beijing, China

**Keywords:** RNA modifications, cyanobacteria, 5-methylcytosine, *Synechocystis* sp. PCC 6803, photosynthesis

## Abstract

RNA modifications have been found in all domains of life and play regulatory roles in diverse biological processes. However, their distribution, function, and regulation in cyanobacteria remain unexplored. Here, we have employed a quantitative RNA profiling strategy based on mass spectrometry analysis to identify 21 different RNA modifications in the model cyanobacterium *Synechocystis* sp. PCC 6803 (*Synechocystis*). Mass spectrometry analyses reveal a dynamic pattern of these RNA modifications under different culture conditions. We subsequently perform transcriptome-wide 5-methylcytosine (m^5^C) profiling in *Synechocystis* by using bisulfite sequencing. In total, we identify 824 high-confidence m^5^C sites in 382 mRNAs, with the majority of m^5^C-modified genes participating in ribosome, RNA degradation, carbon metabolism, and photosynthesis. Combined with the m^5^C-RNA immunoprecipitation detection method, 40.17% (331) m^5^C sites were validated and located within 129 m^5^C-RNA immunoprecipitation peaks on 145 mRNAs. Notably, integrated transcriptomic, proteomic, and m^5^C methylome analysis shows that m^5^C modification is negatively associated with protein abundance and contributes to the RNA–protein discordance, implying the importance of m^5^C on post-transcriptional regulation in *Synechocystis*. Collectively, our study provides a holistic view of RNA modifications and the first mRNA m^5^C map in cyanobacteria, which present a critical database for functional analyses of RNA modifications in cyanobacteria. The method used in this study is applicable to any sequenced prokaryotes and could be applied as a standard part of transcriptomic analysis.

RNA modifications have been found in all domains of life and play regulatory roles in a myriad of physiological processes (1). RNA can undergo modifications across various RNA types, including mRNA, rRNA, tRNA, long noncoding RNA, etc. (2), with over 100 diverse chemical groups known to alter RNA at one or more of its four nucleosides (A, G, C, and U) (3). Modified RNA molecules display distinctive physicochemical properties that have the potential to reshape the three-dimensional structure of RNA (4), thereby impacting molecular interactions and the conformational dynamics (5). Pseudouridine (ψ) was the first RNA modification to be discovered (6), and over the decades, the field of RNA modification has witnessed significant progress and development. Although the precise molecular functions and dynamic regulation of RNA modifications, especially RNA methylation modifications, are not yet fully elucidated, existing research has indicated that these modifications may mediate translation, stability, alternative splicing, transport, and localization processes of RNA molecules (3). In eukaryotes, for example, 5-methylcytosine (m^5^C) modification of tRNA^Leu^ enhances the translation of UUG-rich transcripts, thereby promoting cellular survival under stress conditions (7). In human cells, artificial manipulation of the m^5^C modification system can modulate RNA stability and function, ultimately affecting essential physiological processes, such as cell proliferation and migration (8). Similarly, in zebrafish and mice, Ybx1 regulates mRNA stability through m^5^C modification and plays a crucial role in the expansion of hematopoietic stem and progenitor cells, a key developmental process (9).

The mapping of RNA modifications is a critical part of understanding the function and regulation of these modifications in diverse biological processes. Recent advances in high-throughput sequencing technologies have been used to identify RNA modifications at the transcriptome level (10, 11). Among these, a bisulfite-free detection method known as m^5^C-TAC-Seq was developed to allow direct, base-resolution detection of m^5^C through TET enzyme–assisted oxidation of m^5^C to f^5^C, followed by selective chemical labeling. This approach identifies m^5^C sites *via* pre-enrichment and C-to-T conversion and has been successfully applied in human and mouse cells (12). In *Arabidopsis thaliana*, RNA bisulfite sequencing (Bis-Seq) analysis revealed over 1000 m^5^C sites distributed among mRNAs, long noncoding RNAs, and other noncoding RNAs in varied tissue types. These m^5^C sites intricately govern the stability of tRNA^Asp^ and hold significant importance in orchestrating plant development, potentially in the precise regulation of root length (2). In bacteria, the RNA modification landscape has been relatively under-reported. Studies using m^6^A sequencing technology in *Escherichia coli* and *Pseudomonas aeruginosa* have suggested that m^6^A modification may be closely associated with bacterial respiration, amino acid biosynthesis, and stress response (13). Very recently, Bis-Seq analysis was used to map m^5^C in the model hyperthermophile *Thermococcus kodakarensis* and found that m^5^C is significantly more abundant in this hyperthermophile than in humans (14). To the best of our knowledge, however, no systematic analysis of RNA modifications in cyanobacteria has yet been reported.

Cyanobacteria are essential unicellular bacteria capable of photosynthesis that inhabit various aquatic and terrestrial environments (15). They represent a crucial component of Earth's primary productivity, contributing approximately 50% of the primary production (16) and playing a significant regulatory role in the cycles of oxygen, carbon, and nitrogen (17). Cyanobacteria are considered the precursors of plant chloroplasts, owing to genetic exchanges between plants and cyanobacteria in the endosymbiotic process (18, 19). Their resemblance to plants renders cyanobacteria ideal models for studying photosynthesis and metabolic processes. As a representative among cyanobacteria, *Synechocystis* sp. PCC 6803 (hereafter *Synechocystis*) has been extensively used as a model organism for studies of the mechanisms governing photosynthesis (20) and environmental adaptation (21). In both bacteria and plants, many RNAs undergo appreciable amounts of modifications in response to certain stimuli, and this dynamic process may dictate the fate and activity of the modified RNAs. However, only tRNA modifications in *Synechococcus* *elongatus* PCC 7942 and m^6^A modification in *Synechocystis* have been reported (13, 22), presenting a major obstacle to understanding the functions of RNA modifications in this model organism.

To obtain a comprehensive understanding of RNA modifications in cyanobacteria, we performed a systematic study of RNA modifications in cyanobacteria, where we used *Synechocystis* as a model. We established a quantitative strategy for RNA modifications in cyanobacteria using multiple reaction monitoring (MRM) mass spectrometry (MS) technology. Our findings unveiled the unique types and distributions of RNA modifications in cyanobacteria and shed light on the dynamic regulatory relationship between RNA modifications and stress responses. To elucidate the functions of RNA modifications, we applied Bis-Seq to pinpoint specific m^5^C modification sites in the transcripts of 382 genes in *Synechocystis*. A majority of the m^5^C sites are present on genes involved in ribosome, RNA degradation, carbon metabolism, and photosynthesis. Of these, m^5^C sites in 145 genes were validated using an m^5^C RNA immunoprecipitation (m^5^C-RIP) assay. Notably, our multiomics analyses demonstrated that m^5^Cs play important roles in the regulation of protein abundance in *Synechocystis*. Our study provides a valuable resource for functional analyses of RNA modifications in cyanobacteria. The method and approach can also be used to study RNA modifications in other prokaryotes.

## Experimental procedures

### Cyanobacterial culture and growth conditions

*Synechocystis* was cultivated in BG-11 medium at 30 °C, aerated with filtered air under sustained light exposure (50 μmol·photons·m^–2^ s^–1^) (16, 23). Cells were grown to exponential phase (absorbance at 730 nm = 0.8) and stationary phase (absorbance at 730 nm = 4.7) and then exposed to different stresses, including high light (HL) (500 μmol·photons·m^–2^ s^–1^), high temperature (HT; 42 °C), low temperature (LT; 16 °C), and nitrogen deprivation. For HL treatment, the cultures grown to the exponential phase were subjected to the HL condition for 60 min. For different temperature treatments, cells were grown to the exponential phase and immediately subjected to HT and LT conditions for 30 min. For nitrogen deprivation, the cultures grown to the exponential phase were collected and resuspended nitrogen-deprived BG11 medium for another 12 h.

### RNA extraction and digestion

The *Synechocystis* cultures grown under various conditions were harvested by centrifugation and washed three times with diethyl pyrocarbonate–treated water. Subsequently, the collected cells were then ground into powders using liquid nitrogen and then resuspended in 1 ml TRIzol reagent (Ambion). The supernatant was collected, and 200 μl of chloroform solution was added. After centrifuging at 12,000*g* for 15 min, the upper layer containing RNA was carefully collected, and 400 μl of isopropanol was subsequently added for RNA precipitation. The RNA precipitate underwent two washes with 1 ml of 75% ethanol, and its purity was assessed by measuring the absorbance at 260 nm/absorbance at 280 nm using a NanoDrop 2000 spectrophotometer (Thermo Fisher Scientific). The RNA concentration was determined using Qubit RNA BR Assay Kits (Q10210; Thermo Fisher Scientific).

### Identification and quantitation of RNA modifications by LC–MS/MS

Total RNA samples were digested using Nucleoside Digestion Mix Kit (M0649S; NEB) at 37 °C for 1 h and analyzed by an ACQUITY UPLC system coupled to a Xevo TQ MS (Waters). The digested nucleosides were separated with an analytical C_18_ LC column (1.7 μm particle size, 100 mm × 2.1 mm; Waters) and eluted with a 12 min gradient of an increase from 1% to 12% of solvent B (0.1% formic acid/90% acetonitrile, v/v) over 4 min, 12% to 70% in 3 min, 70% to 1% in 3 min, and holding at 1% for the last 6 min at 0.2 ml/min flow rate. The mass spectrometer was operated in MRM mode. The column temperature was maintained at 30 °C, and a capillary voltage was set to 3 kV, and a cone voltage was optimized to 40 V. High nitrogen drying gas was used for nebulization and drying, with a nitrogen gas flow rate of 800 L/h and a desolvation temperature of 450 °C. MassLynx V4.1 (Waters) software was utilized for peak area quantification and data processing (24). Details of the synthesized 42 nucleoside standards are provided in Table S1. The other compound-dependent parameters for these standards are listed in Table S2.

### RNA-Seq and data analysis

Total RNA samples were isolated from *Synechocystis* in exponential and stationary growth phases. The rRNA was removed using Ribo-off rRNA Depletion Kit Mega (Bacteria) (N417-01; Vazyme) according to the manufacturer’s protocol. Briefly, the total RNA samples were incubated with the rRNA hybridization probe, followed by RNase H and DNase I digestion, ultimately removing rRNA from the total RNA. The products were purified with VAHTS RNA Clean Beads to remove salts and buffer concentrates. Subsequently, RNA libraries were constructed using VAHTS Universal V6 RNA-Seq Library Prep Kit (NR604-01; Vazyme) according to the manufacturer’s instructions, and the library quality was evaluated using the BioAnalyzer 2100 system (Agilent Technologies, Inc). Finally, sequencing was performed on an Illumina NovaSeq 6000 platform with 150 bp paired-end reads. Low-quality reads and adapter sequences were first trimmed using Cutadapt software (version 1.9.3) (25). The clean reads were then aligned to the *Synechocystis* genome (GCF_000009725.1_ASM972v1) with HISAT2 software (v2.0.5) (26). StringTie software (v2.2.1) (27) was used to calculate fragments per kilobase of transcript per million mapped read (FPKM) values. The gene is considered expressed if the FPKM value was detected in three biological replicates.

### RNA Bis-Seq

Given that rRNA constitutes >95% of total RNA in prokaryotes (28), its overwhelming abundance severely compromises the detection of modification sites during sequencing. The purpose of this study was to identify as many m^5^C modifications as possible and comprehensively map functional m^5^C sites in *Synechocystis*, thereby the rRNA was removed from the total RNA using the RiboPOOL rRNA depletion kit (dp-K024-71; siTOOLs Biotech, Inc) according to the manufacturer’s protocol. Briefly, the total RNA samples were hybridized with the streptavidin-coated magnetic beads to remove the rRNA. The RNA samples were then purified with silica-based RNA clean-up column to remove salts and buffer concentrates. Subsequently, the rRNA-depleted RNA was subjected to bisulfite conversion and purification using the EZ RNA Methylation Kit (R5002; Zymo Research, Inc), according to the manufacturer’s protocol (https://files.zymoresearch.com/protocols/_r5001_r5002_ez_rna_methylation-_kit.pdf). Briefly, to test the conversion efficiency, an unmodified positive RNA spike-in control was added into the RNA samples (Table S3). rRNA-depleted RNA was then mixed with RNA Conversion Reagent in a PCR tube and kept at 70 °C for 5 min, followed by 54 °C for 45 min. After cooling to room temperature, the mixtures were loaded into the Zymo-Spin IC Column for desulphonation. After washing several times, the bisulfite-converted RNA samples were eluted with DNase/RNase-Free Water and collected by centrifugation. RNA libraries were constructed utilizing the NEBNext Ultra II Directional RNA Library Prep Kit (E7760L; New England BioLabs, Inc), and the library quality was evaluated using the BioAnalyzer 2100 system (Agilent Technologies, Inc). Finally, sequencing was performed on an Illumina NovaSeq 6000 sequencer with 150 bp paired-end reads.

### RNA m^5^C-RIP and data analysis

According to the manufacturer's protocol, experiments were performed using GenSeq m^5^C MeRIP Kit (GS-ET-003). Briefly, 180 μg of total RNA was first fragmented and then dissolved in DNase/RNase-Free Water. A 3 μg aliquot of the fragmented RNA was reserved as the input group for RNA-Seq, whereas the remaining RNA was kept on ice for subsequent steps. The RNA was mixed with preprepared magnetic beads and incubated at 4 °C for 1 h with rotation. The beads were washed multiple times with 1× immunoprecipitation (IP) buffer, followed by incubation at 55 °C with rotation and mixing for 45 min. The immunoprecipitated beads were then placed on a magnetic stand, and 50 μl of supernatant was transferred to 150 μl of preprepared MS magnetic beads. Absolute ethanol (200 μl) was added, and the mixture was incubated with rotation for 5 min. The beads were subsequently washed multiple times with 75% ethanol, resuspended in nuclease-free water, and the supernatant containing RNA fragments was collected. The rRNAs from IP and input samples were removed using Ribo-off rRNA Depletion Kit Mega (Bacteria) (N417-01; Vazyme) according to the manufacturer’s protocol. RNA libraries were constructed using the NEBNext Multiplex Small RNA Library Prep Set for Illumina (New England Biolabs, Inc), and library quality was assessed using the BioAnalyzer 2100 System (Agilent Technologies, Inc). Finally, sequencing was performed on an Illumina NovaSeq X Plus platform with 150 bp paired-end reads. Low-quality reads and adapter sequences were first trimmed using the Cutadapt software (version 1.9.3) (25). The clean reads were then aligned to the *Synechocystis* genome (GCF_000009725.1_ASM972v1) with HISAT2 software (version 2.0.5) (26). m^5^C-RIP-Seq peaks were then detected using MACS3 peak caller (29) on pooled IP *versus* input, according to the user manual. Peaks were considered if *p* < 0.00001, *q* < 0.01, and fold change >2 (30).

### Determination of cytosine conversion rate and m^5^C profiling

Paired-end reads were harvested and quality controlled based on a Q score of Q30. Low-quality reads and adapter sequences were removed using Cutadapt software (version 1.9.3) (25). The clean reads were then aligned to the *Synechocystis* genome (GCF_000009725.1_ASM972v1) combined with an unmodified RNA spike-in control sequence using meRanGh (a component of meRanTK) software (version 1.3.0) with default parameters (31). First, the reference database (including the Synechocystis genome and control sequence) was converted into a bisulfite-specific index with the command: meRanGh mkbsidx -t 10 -fa *Synechocystis*.fa -id./*Synechocystis*.BS.IDX. The clean Bis-Seq reads were then aligned to the reference database by meRanGh using the created bisulfite database index with the following command: meRanGh align -o./WT.m5c/meRanGhResult -f./WT.m5c_R2.fastq.gz -r./WT.m5c_R1.fastq.gz -t 20 -S BS_WT.m5c.sam -ud./WT.m5c/meRanGhUnaligned -un -MM -id./*Synechocystis*.BS.IDX -GTF *Synechocystis*.gtf -bg -mbgc 10 -mbp. To confirm the bisulfite conversion rate, the meRanCall (a component of meRanTK) software was applied to assess the conversion rate of the positive control sequence using the following command: meRanCall -p 32 -s RNA-BSseq_sorted.bam -f./*Synechocystis*.fa -ccr -c SeqID UnMethylated_Control, according to the user manual. The methylation status of cytosine within the *Synechocystis* genome was extracted by meRanCall software using the following command: meRanCall -p 32 -o./meRanCallResult.txt -bam RNA-BSseq_sorted.bam -f *Synechocystis*.fa -rl 150 -sc 10 -md 1 -mr 0.01 -ei 0.1 -cr 0.99 -bed63 -np -gref. The methylation calls allowed a maximum of one read duplicate per position (-md 1) and a minimum of 0.1 methylation ratio of a single C (-mr 0.01) to consider a cytosine methylated. The error interval for *p* value calculation of methylation level was set to 0.1 (-ei 0.01), and the C to T conversion rate was set to 0.99 (-cr 0.99). Finally, the m^5^C sites were considered credible with a methylation level (methRate) of ≥0.1 and coverage depth of ≥10 reads at each site, as previously described (32, 33).

### Protein extraction and trypsin digestion

*Synechocystis* strains in exponential and stationary growth phases were harvested and washed three times with PBS buffer (0.137 M NaCl, 2.7 mM KCl, 1.8 mM KH_2_PO_4_, and 0.01 M Na_2_HPO_4_). Cells were subsequently resuspended with ice-cold lysis buffer (150 mM NaCl, 20 mM Tris–HCl [pH 7.5], 1% Triton X-100, and 1 mM PMSF) and disrupted by sonication (3 s on, 3 s off) for 30 min on ice using a JY92-IIN sonicator (JY92-IIN; Ningbo Scientz Biotechnology Co, Ltd). Cellular debris was removed by centrifugation at 5000*g* for 20 min at 4 °C. The resulting supernatants were collected, and protein concentration was determined by a BCA assay kit (P0012; Beyotime). Whole cell lysates were reduced, alkylated, and trypsin digested as described previously (34). Briefly, protein lysates from three biological replicates were precipitated using 80% (v/v) ice-cold acetone, followed by dissolution in 50 mM ammonium bicarbonate. Extracts were reduced with 25 mM DTT (D9779; Sigma–Aldrich) at 37 °C for 45 min and alkylated with 50 mM iodoacetamide (I1149; Sigma–Aldrich) at room temperature for 10 min in the dark. Protein samples were then digested with trypsin (V5111; Promega) to a protein mass ratio of 1:100 at 37 °C for 24 h. The digestion was quenched by adding 0.1% (v/v) TFA and desalted using a self-packed C_18_ STAGE column (C_18_, 40 μm; Agilent Technologies). Peptides were dried with a vacuum centrifuge and stored at −80 °C for further use.

### LC–MS/MS analysis

The peptide samples were dissolved in solution A containing 0.1% TFA and 2% acetonitrile and then analyzed using an online nanoEASY1200 HPLC system (Thermo Fisher Scientific) coupled with an Orbitrap Q Exactive HFX mass spectrometer (Thermo Fisher Scientific). Peptides were separated using a 75 μm × 15 cm C_18_ column (C_18_, 2 μm; Thermo Fisher Scientific) and eluted with a gradient of 5% to 80% solvent B (90% acetonitrile and 0.1% formic acid) at a flow rate of 300 nl/min for 125 min as follows: 0 to 1 min, 3% to 6% (v/v) solvent B; 1 to 61 min, 6 to 17% (v/v) solvent B; 61 to 86 min, 17% to 23% (v/v) solvent B; 86 to 105 min, 23% to 32% (v/v) solvent B; 105 to 115 min, 32% to 38% (v/v) solvent B; 115 to 116 min, 38% to 95% (v/v) solvent B; and holding at 95% (v/v) solvent B for the last 9 min. The eluted peptides were ionized and detected using the Orbitrap Q Exactive HFX mass spectrometer (Thermo Fisher Scientific). MS data acquisition was carried out in a data-independent acquisition (DIA) model using 80 variable windows covering a mass range of 300 to 1800 at a resolution of 60,000. The maximum injection time for full MS was 20 ms, and the automatic gain control target was set to 3E6. Following every survey scan, 80 DIA scans were used with a specific predefined inclusion list, which contained the exact mass of the selected precursor ion and isolation window (Table S4). Precursor ions were fragmented with a high-energy collision dissociation collision energy with a normalized collision energy of 28% and analyzed in the Orbitrap at a resolution of 30,000 at 200 *m/z*. Automatic gain control target for MS/MS was set to 1E6.

### Protein identification and quantification

DIA data were processed with DIA-NN (version 1.8.1) (35) using a library-free workflow against the *Synechocystis* protein database (http://genome.annotation.jp/cyanobase). “FASTA digest for library free search/library generation” and “Deep learning spectra, RTs and IMs prediction” options were used for precursor ion generation. The protease was set to trypsin/P with two maximum missed cleavages. Carbamidomethylation (Cys) was set as a fixed modification, whereas oxidation (Met) and acetylation (protein N-terminal) were set as dynamic modifications. The maximum number of variable modifications was set to 1. Peptide length was set to 7 to 30 amino acids, and the precursor charge range was restricted to 1 to 4. The false discovery rate at the precursor, peptide, and protein levels was set to 1%. Label-free quantification protein intensities were extracted from the DIA-NN report file “report.pg_matrix.tsv.” Only proteins that had to be detected in three biological replicates were considered expressed.

### Bioinformatics analysis

Sequence motif analysis was performed by the pLogo tool (https://plogo.uconn.edu/) and the DREME tool (https://meme-suite.org/meme/tools/dreme). According to the manufacturer’s protocol of pLogo, we extracted prealigned sequences spanning modified nucleotides (10 nucleotides upstream and downstream of the modified site) as foreground data, and the whole-genome background was selected as background data to eliminate confounding biases from gene-specific features, ensuring the detected motifs reflect intrinsic enzyme specificity. The distribution of m^5^C in *Synechocystis* was graphically depicted using the Tbtools II plugin Circos (36, 37). Gene Ontology (GO) annotation of all identified proteins was performed by using InterProScan (version 5.61-93.0) software (38). Kyoto Encyclopedia of Genes and Genomes (KEGG) enrichment analysis was conducted using the KOBAS (version 2.0) software (39) and visualized by using the ggplot2 (version 3.4.3) package. Clusters of orthologous group (COG) annotation was performed using the eggNOG-mapper software (40). Protein–protein interaction (PPI) network analysis was conducted using the STRING database (41). The network was visualized by Cytoscape software (version 3.9.1) (42) and further analyzed for densely connected regions using the Molecular Complex Detection (MCODE) algorithm in Cytoscape (National Resource for Network Biology). The heatmaps were generated using the pheatmap package (version 1.0.12). The correlation analysis between protein and mRNA abundances was performed using the R framework (version 4.3.1) with the Spearman's correlation method. The protein-to-mRNA ratio (PTR) was calculated as the ratio of protein abundance to the corresponding mRNA abundance for each gene (43).

## Results

### Global RNA modification discovery in *Synechocystis*

**Figure 1:**
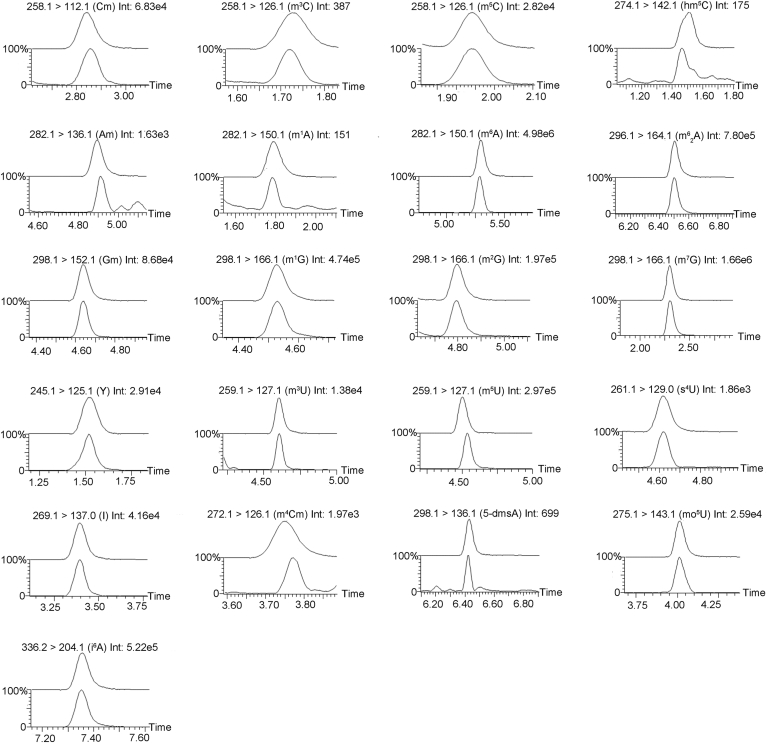
**Comparison of the extracted ion chromatograms (EICs) between modified nucleoside standards and RNA samples from *Synechocystis*.** The *upper panel* represents the EICs of modified nucleoside standards, whereas the *lower panel* shows the EICs of *Synechocystis* RNA samples. *X*-axis: retention time; *Y*-axis: percentage of base peak intensity (%). Labels above each EIC indicate the precursor/product ions, modification type, and integrated peak area determined by mass spectrometry.

**Figure 2:**
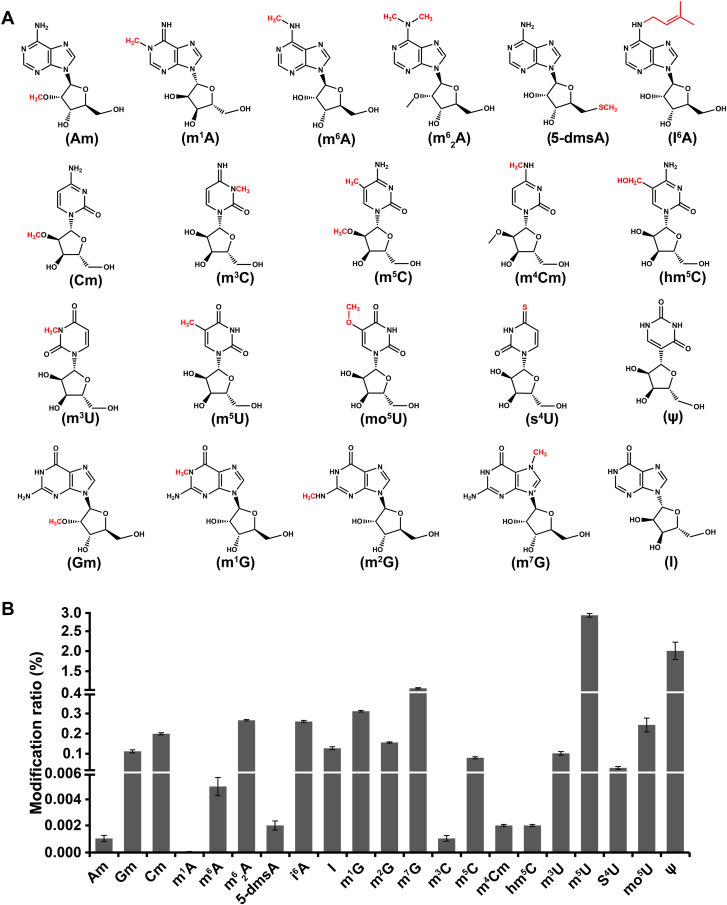
**Quantitative detection of modified ribonucleosides in *Synechocystis*.***A,* chemical structures of the identified modified ribonucleosides in *Synechocystis*. *B,* LC–MS/MS quantification of the identified modified ribonucleosides in *Synechocystis*. Data presented as means ± SD from four biological replicates.

To identify the types and abundance of RNA modifications in *Synechocystis*, we synthesized 42 common nucleoside standards (Table S1) and established an MRM-based MS approach for the identification of modifications on different nucleosides in RNA (Table S2), including the nucleosides, precursor and product ions, cone potential, collision energy, and retention time. Figure S1 presents the MS/MS spectra of the identified 42 common nucleoside standards, exhibiting the characteristic fragment ions for each standard. The fragment ions of most standards are consistent with the data from the PubChem website (https://www.ncbi.nlm.nih.gov/pccompound), confirming the accuracy and reliability of our MS approach (Fig. S1). Although reference spectra were unavailable on PubChem for eight nucleoside standards (8-oxoA, m^1^I, m^7^s^6^G, I^5^C, hm^5^U, m^6^G, m^4^Cm, and ho^5^U), we performed fragmentation analyses using structural information and established MS fragmentation principles. In general, our results indicate that the glycosidic bond (C-N bond) between the ribose and base within nucleoside molecules undergoes cleavage, leading to the separation of the base from the ribose and the generation of corresponding base fragment ions and ribose fragment ions (44). For the diagnostic base fragment, 8-oxoA, bruised by an oxygen atom at C-8, snaps its glycosidic bond with almost audible relief; the oxidized adenine that tumbles free carries the unmistakable signature of reactive oxygen species attack at *m/z* 152.0588 (C_5_H_6_N_5_O_2_^+^), a mass that can arise from no other lesion in the nucleic acid lexicon. m^1^I, quieter but just as specific, leaves behind a methylated hypoxanthine whose *m/z* 151.0604 (C_6_H_7_N_4_O_2_^+^) speaks of a single methyl quietly parked on N^1^, distinguishing it from every other inosine adduct. m^7^s^6^G carries two badges of honor, an N^7^-methyl and a 2-thio group located on the guanine scaffold. When the ribose departs, the doubly modified guanine appears at *m/z* 182.0900 (C_6_H_9_N_5_S^+^), the sulfur isotope envelope confirming that the thiocarbonyl is still intact. I^5^C refuses to let go of its isopentenyl tail; instead, the entire cytokinin-like base escapes at *m/z* 238.1304 (C_10_H_16_N_5_^+^), the extra 10 carbons and 15 hydrogens betraying the prenyl side chain that defines this epitranscriptomic mark. hm^5^U opts for a different narrative, retaining the ribose, whereas the 5-hydroxymethyl group remains covalently linked; the resulting intact nucleoside ion at *m/z* 275.1240 (C_10_H_15_N_2_O_6_^+^) carries both sugar and modification, a subtle but unmistakable fingerprint for hydroxymethylated uridine. m^6^G, discreet yet decisive, yields N^2^-methylguanine at *m/z* 166.0723 (C_6_H_8_N_5_O^+^), a mass increment of only 14 Da but one that places the methyl precisely where base-excision glycosylases will later look. Finally, ho^5^U departs as the minimalist among the group, shedding sugar and all else save the 5-hydroxyuracil that registers at *m/z* 129.0402 (C_4_H_5_N_2_O_3_^+^), a fragment so light that only the extra oxygen at C-5 distinguishes it from canonical uracil. For m^4^Cm, owing to the presence of a methylation group on the N^4^ of the pyrimidine ring, the diagnostic base fragment of N^4^,2-O-dimethylcytosine at *m/z* 126.08 (C_6_H_10_N_3_O^+^) can be detected and therefore serves as a specific marker for the presence of m^4^Cm. The exact mass and isotopic pattern of each fragment make it an exquisitely specific sentinel for its parent lesions, unambiguously supporting its identification in the analytical workflow (Fig. S1). Subsequently, total RNA from *Synechocystis* was extracted and completely digested to mononucleosides, which were then subjected to analysis by MS. We compared the extracted ion chromatogram retention times and the characteristic MS/MS fragment ions of the RNA modifications from *Synechocystis* samples with those of standards, consistent with methodologies reported in previous studies (45). As shown in Figure 1, the retention times of *Synechocystis* nucleosides could match well with those of standards. Furthermore, the MS/MS spectra of these RNA modifications in *Synechocystis* RNA samples alongside those of the respective standards (Fig. S2). Through comparative analysis of the extracted ion chromatograms and MS/MS spectra of *Synechocystis* RNA samples with those of standards, we ultimately identified 21 RNA modifications in *Synechocystis* samples, whose retention times and characteristic fragment ions showed high consistency with the standards. Figure 2*A* showed the structure of identified 21 RNA modifications, encompassing 2′-O-methylation modifications (Am, Cm, and Gm) and 5-dmsA on ribose, monomethylation modifications (m^1^A, m^6^A, m^6^_2_ A, m^3^U, m^5^U, m^4^Cm, m^3^C, m^5^C, m^1^G, m^2^G, and m^7^G) on bases, as well as other chemical modification groups on RNA (i^6^A, hm^5^C, s^4^U, mo^5^U, I, and ψ). Further quantitative analysis of these modifications revealed that the highest content of these modifications was on U with methylation (2.897% m^5^U/rU), followed by the ψ (2.008% ψ/rU), and G with methylation (1.075% m^7^G/rG) also showed relatively high abundance in *Synechocystis* RNA (Fig. 2*B*). Although m^6^A is widely distributed in eukaryotes, its content is relatively low in bacteria, especially in cyanobacteria, where it is less than 0.04% (m^6^A/rA) (13). Consistently, we observed the presence of m^6^A in *Synechocystis* RNA, whose m^6^A/rA ratio was about 0.005% (Fig. 2*B*). These results clearly demonstrate the widespread occurrence of diverse RNA modification types within cyanobacteria, implying that these RNA modifications may have distinct functional roles and possess intricate regulatory functions in *Synechocystis* cellular process.

### RNA modifications respond to stress treatments in *Synechocystis*

**Figure 3:**
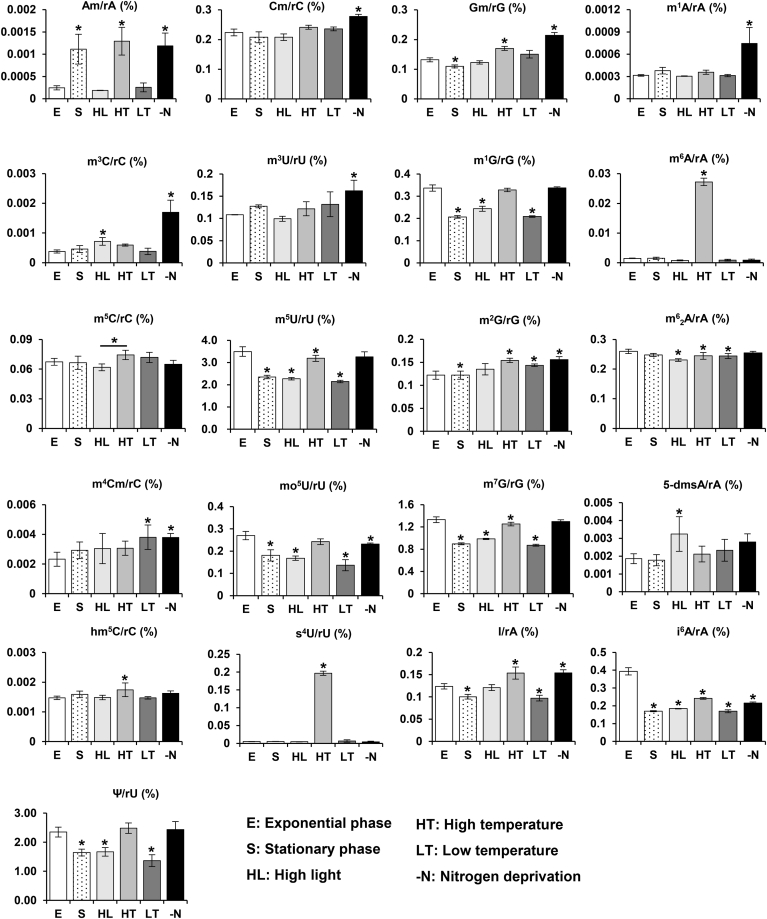
**Quantitative analysis of RNA modifications in *Synechocystis* under different growth conditions.** Each modification ratio was calculated based on nucleoside levels under various conditions: exponential phase (E), stationary phase (S), high light (HL), high temperature (HT), low temperature (LT), and nitrogen deprivation (-N). Error bars represent the standard error of the mean from three biological replicates. Statistical significance was determined using ANOVA (∗*p* < 0.05).

For the quantitative analysis of modified nucleosides in *Synechocystis* RNA, total RNA was extracted from *Synechocystis* cultivated under various growth conditions, completely digested to mononucleosides, and quantified using an established MRM-based MS approach. In this work, we selected the stress treatments that mimicked native conditions experienced by *Synechocystis* for further analysis, including exponential phase (E), stationary phase (S), HL, HT, LT, and nitrogen deprivation (-N) conditions. Finally, the 21 RNA modifications exhibited dynamic changes across different conditions (Fig. 3), based on ANOVA (*p* < 0.05 for all comparisons). Compared with the exponential growth phase, the ratios of Am/rA, m^2^G/rG increased significantly in the stationary phase, whereas Gm/rG, i^6^A/rA, I/rA, m^1^G/rG, m^7^G/rG, m^5^U/rG, mo^5^U/rU, and ψ/rU were detected at relatively lower levels in the stationary phase. Under HL, levels of m^6^_2_A/rA, i^6^A/rA, m^1^G/rG, m^7^G/rG, m^5^U/rU, mo^5^U/rU, and ψ/rU were significantly reduced. Similarly, LT conditions led to a decrease in i^6^A/rA, m^6^_2_A/rA, I/rA, m^1^G/rG, m^7^G/rG, m^5^U/rU, mo^5^U/rU, and ψ/rU, whereas HT treatment also reduced an obvious decrease of i^6^A/rA, m^6^_2_A/rA, m^7^G/Rg, and m^5^U/rU. Conversely, the level of m^3^C/rC and 5-dmsA/rA increased under HL conditions, and the levels of m^6^A/rA, s^4^U/rU, m^4^Cm/rC, hm^5^C/rC, and m^2^G/rG exhibited remarkable increase under HT or LT conditions. In the situation of nitrogen deprivation, the levels of Am/rA, Gm/rG, Cm/rC, m^1^A/rA, I/rA, m^2^G/rG, m^3^C/rC, m^4^Cm/rC, and m^3^U/rU were upregulated, whereas the modifications of i^6^A/rA and mo^5^U/rU were downregulated. Together, these results demonstrate a dynamic abundance of these RNA modifications under various stress conditions, suggesting a unique regulatory mechanism in response to diverse environmental stresses.

It is well known that m^6^A is the most abundant internal modification in eukaryotic mRNAs and has been recently investigated in diverse model bacterial species (13). However, consistent with the other bacteria, the abundance of m^6^A in cyanobacteria showed extremely low levels in various stress treatments. In contrast, m^5^C is also among the most prevalent post-transcriptional modifications in eukaryotic mRNAs. In photosynthetic organisms, m^5^C is widely distributed and plays an important role in gene regulatory networks underlying plant development (30). In the present work, m^5^C also exerts a certain influence on *Synechocystis*.

### Bis-Seq reveals abundant and high-confidence m^5^C modification in the *Synechocystis* transcriptome

**Figure 4:**
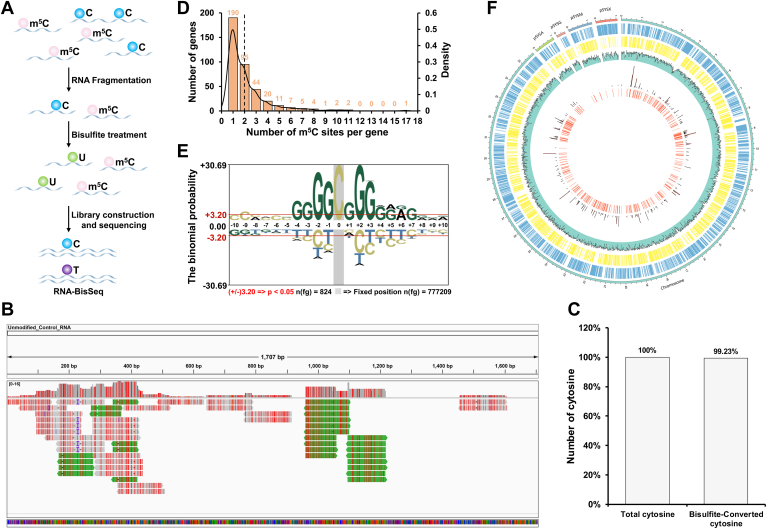
**5-Methylcytosine (m^5^C) methylome in *Synechocystis*.***A,* schematic diagram of the RNA bisulfite sequencing (Bis-Seq) strategy. This strategy encompassed RNA fragmentation, bisulfite treatment, library construction, and sequencing. *B,* visual inspection of reads aligned to the unmodified positive control sequence using Integrative Genomics Viewer. These aligned reads were used to calculate the bisulfite conversion rate in subsequent analyses. *C,* bisulfite conversion efficiency of the unmodified positive control sequence. The conversion efficiency was determined by calculating the percentage of bisulfite-converted cytosines relative to the total of cytosines in the sequence. *D,* histogram showing the distribution of m^5^C sites per gene. *E,* sequence motif analysis of m^5^C sites in *Synechocystis*. Ten nucleotides upstream and downstream of each modified site were used as foreground data, and the entire genome served as background to control for compositional bias. *F,* Circos plot depicting m^5^C methylation sites in *Synechocystis*. *Circles*: 1. Chromosomes and plasmids; 2. Genes on the plus strand; 3. Genes on the minus strand; 4. GC ratio; 5. Number of m^5^C sites; and 6. m^5^C methylated genes.

Bisulfite treatment converts nonmethylated cytosine into uracil, whereas m^5^C remains unchanged during the treatment. Cytidines retained after bisulfite treatment are therefore reported to be m^5^C. Bisulfite treatment followed by sequencing permits the identification of m^5^C modifications at single-nucleotide resolution (Fig. 4*A*). Given that rRNA constitutes >95% of total RNA in prokaryotes (28), its overwhelming abundance severely compromises the detection of modification sites during sequencing. To achieve a transcriptome-wide landscape of m^5^C at single-nucleotide resolution and identify as many m^5^C modifications as possible, we performed RNA Bis-Seq on ribosomal-depleted RNA from *Synechocystis* by using the EZ RNA Methylation Kit from Zymo Research (Fig. 4*A*). The ability to detect m^5^C in RNA efficiently and accurately has been troublesome because the RNA with secondary structures or double-stranded regions may resist bisulfite conversion and cause false-positive detection of m^5^C sites (46). To solve these problems, the kit used in this study has been optimized and validated for bisulfite conversion of RNA. After performing bisulfite treatment, more than 99% of nonmethylated C residues are converted to U with >99% protection of m^5^C. To examine cytidine conversion rates, we analyzed the conversion efficiency of the control sequence that was spiked into our RNA sample. Figure 4*B* showed the Integrative Genomics Viewer displaying the mapping of RNA-Seq reads from the unmodified positive control sequence. Using the meRanCall tool, we extracted reads from the control sequence and calculated the bisulfite conversion rate (31). As expected, 99.23% of unmodified cytosine in the positive control was converted (Fig. 4*C*), confirming near-complete C-to-U conversion, which allows accurate identification of m^5^C sites in our RNA samples. The bisulfite-converted RNA was then used to construct libraries for sequencing. After removing low-quality reads and adapter sequences, the clean reads were aligned to the reference genome of *Synechocystis* concatenated with an unmodified positive control sequence. Based on our Bis-Seq data, we identified 824 high-confidence m^5^C sites across mRNA from 382 genes in *Synechocystis* (Table S5). Among these identified sites, the median number of m^5^C sites per gene was 2, with five genes harboring more than 10 m^5^C sites. For instance, the polynucleotide phosphorylase (encoded by *sll1043*) possessed the highest number of m^5^C sites (15), 11 m^5^C sites were observed in phytochrome (*slr0473*) and phosphoenolpyruvate synthase (*slr0301*), as well as 10 sites in ribonuclease E (*slr1129*) and DNA-directed RNA polymerase subunit beta (*sll1789*). These findings indicated the important roles of m^5^C in RNA metabolism, photosynthesis, and metabolism. Furthermore, nearly half of the genes had a single m^5^C site, whereas most genes harbored between two and eight modification sites (Fig. 4*D*).

To investigate whether the identified m^5^C sites share common sequence elements that are characteristic of m^5^C RNA modification, we performed an unbiased search for consensus motifs enriched around the m^5^C sites. As illustrated in Figure 4*E*, the base G was most significantly enriched in positions spanning −4 to +7 around the m^5^C modifications. While m^5^C sites have previously been reported to localize in a G-rich environment (G at +1, +2, +3, and +4 positions) (47), our findings revealed a distinct G-rich motif in *Synechocystis*. Specifically, the base G was significantly enriched at positions −4 to −1, A at +6, and C at −9 (Fig. 4*E*). This unique G-rich pattern suggests that m^5^C modifications in cyanobacteria may be associated with specific motif sequences, potentially recognized by cyanobacterial proteins and serving specialized biological functions. We also constructed a circular methylome map of m^5^C in cyanobacteria to display the global distribution of all identified sites mapped to chromosomes and plasmids (Fig. 4*F*). Despite the majority of modification sites being located on chromosomes, there are still five sites that are located on plasmids, including two-component response regulator, chromate transporter, sulfide–quinone reductase, WD repeat–containing protein, and other hypothetical proteins.

### Validation of m^5^C modification by m^5^C-RIP sequencing

**Figure 5:**
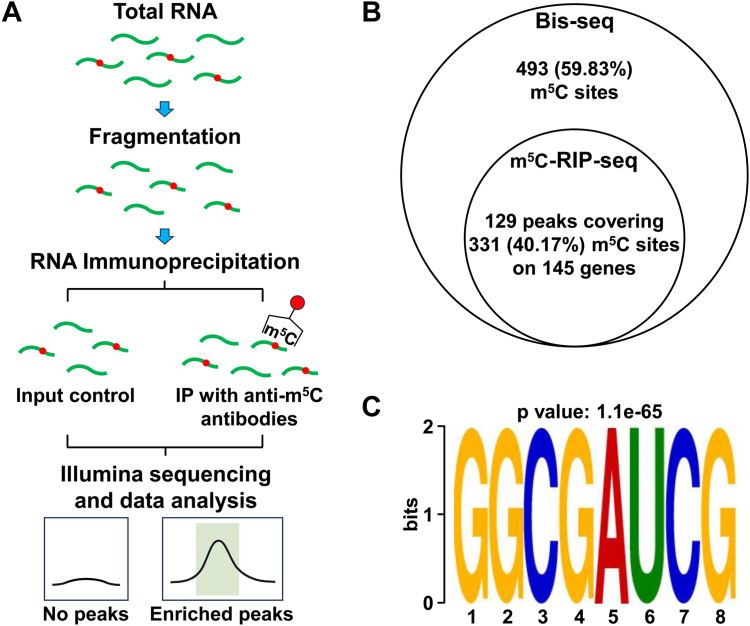
**Validation of 5-methylcytosine (m^5^C) modification by m^5^C-RIP in *Synechocystis*.***A,* workflow of m^5^C-RIP, including RNA extraction, RNA fragmentation, RNA immunoprecipitation, library construction, sequencing, and data analysis. *B,* Venn diagram showing overlapping m^5^C peaks and sites identified by both bisulfite sequencing and m^5^C-RIP. *C,* leading motif at putative m^5^C sites identified with m^5^C-RIP-Seq. Motifs and associated *p* values were generated using DREME software. RIP, RNA immunoprecipitation.

To validate the m^5^C methylome derived from Bis-Seq, we performed m^5^C-RIP sequencing, the most widely used method for transcriptome-wide m^5^C mapping (48), using RNA extracted from *Synechocystis* cells. As shown in Figure 5*A*, total RNA was immunoprecipitated with an anti-m^5^C antibody, whereas untreated RNA served as the input control. Finally, a total of 372 m^5^C peaks were detected in this assay (Table S6). To further confirm the m^5^C sites detected by Bis-Seq, we compared them with the m^5^C-RIP peaks. Among the 824 m^5^C sites identified by Bis-Seq, 40.17% (331) m^5^C sites were located within 129 m^5^C-RIP peaks on 145 genes (Fig. 5*B* and Table S7), which is similar to the observation in other organisms (49, 50). The remaining 493 sites (59.83%) were uniquely detected by Bis-Seq, suggesting that Bis-Seq provides higher sensitivity and single-nucleotide resolution for m^5^C detection. The identified m^5^C modifications were subsequently subjected to DREME motif analysis, and several motifs were significantly enriched among the identified m^5^C sites (Fig. S3). The leading consensus sequence is GGCGAUCG (*p* = 1.1e-65) (Fig. 5*C*), found at 52.15% (194) of identified m^5^C positions in *Synechocystis* RNAs. This indicates that the occurrence of m^5^C exhibits a certain preference for the base sequence of RNA. Collectively, these results demonstrate that m^5^C-RIP sequencing supports the reliability of our bisulfite-based m^5^C methylome data and further confirms the presence of conserved sequence preferences associated with m^5^C modification in *Synechocystis*.

### Functional characterization of m^5^C-modified genes in *Synechocystis*

To further uncover potential functional impacts of m^5^C in *Synechocystis*, we performed GO functional annotations for all the identified 382 m^5^C-methylated genes according to their biological process, cellular component, and molecular function. From the perspective of biological processes, the m^5^C-methylated genes were predominantly involved in amino acid and peptide biosynthetic processes (*p* = 1.79E-09, *p* = 4.11E-09), translation (*p* = 6.18E-09), gene expression (*p* = 2.61E-04), along with protein folding (*p* = 2.68E-02). Moreover, a substantial array of genes was associated with dicarboxylic acid metabolic process (*p* = 2.68E-02), carboxylic acid biosynthetic process (*p* = 2.70E-02), and photosynthesis (*p* = 3.60E-02) (Fig. S4*A* and Table S8). These findings indicated that m^5^C modifications may have significant impacts on amino acid and peptide translation, and photosynthesis in *Synechocystis*. Consistently, analysis of molecular function showed that most genes were significantly enriched in oxidoreductase activity (*p* = 1.02E-04) and carbon–oxygen lyase activity (*p* = 2.07E-02), which were related to metabolism. The other prevalent enrichment occurred in molecular function linking with structural constituent of ribosome (*p* = 1.49E-13), translation regulator activity (*p* = 5.93E-04), electron transfer activity (*p* = 3.94E-03), and proton transmembrane transporter activity (*p* = 4.83E-02), suggesting the potential functional impacts in protein translation and photosynthesis (Fig. S4*B* and Table S8). The enrichment analysis of subcellular localization further revealed that genes associated with the ribosome, ribonucleoprotein complex, thylakoid, and photosynthetic membrane were mostly over-represented in our data (Fig. S4*C* and Table S8). COG analysis was carried out to infer that m^5^C-regulated genes in cyanobacteria might undergo similar regulatory patterns in their evolutionarily conserved orthologs across species. We next classified these genes into a wide range of functional classes using the COG database, identifying a broad range of functions. As expected, a large portion of genes were related to translation, ribosomal structure and biogenesis, energy production and conversion, and amino acid transport and metabolism (Fig. S5 and Table S9).

PPI network analysis can serve as an alternative strategy to analyze physical and functional interactions. To further obtain a global view of the m^5^C-modified gene regulatory network, we conducted an interaction network of all 382 m^5^C-modified genes using the STRING database, and the interaction map consisted of a large network covering 186 modified genes (Table S10). Subsequently, this interaction network was further analyzed for densely connected regions using a graph theoretic clustering algorithm, MCODE (51), which is part of the plug-in toolkit of network analysis and visualization software Cytoscape. The MCODE algorithm assigns weights to nodes based on the density of their local neighborhoods and performs outward traversal starting from locally dense seed proteins. Guided by predefined parameters, it isolates densely connected regions within PPI networks, which potentially correspond to molecular complexes (51). We applied the MCODE clustering algorithm to identify tightly interconnected modules and retrieved a highly interconnected network comprising 86 modified genes. The top four interconnected clusters consisted of several genes involved in photosynthesis, ribosome, and the NDH complex systems (Fig. S6 and Table S10). Among these connected genes, three genes (*pnp*, *rpoC2*, and *rne*) containing 17, 10, and 10 m^5^C modification sites, respectively, exhibited high interactions with ribosome-related genes, implying the potential functional impacts of m^5^C modification in protein translation. Consequently, we speculated that m^5^C modification may play an important regulatory role in protein translation, photosynthesis, and metabolism.

To explore the potential functions of m^5^C-modified genes, we performed KEGG pathway enrichment analysis to examine whether these regulated genes are clustered in specific pathways to function collectively. Consistent with the results of GO functional annotation, our enrichment analysis revealed that the m^5^C-modified genes were markedly enriched in ribosome (*p* = 5.60E-11), photosynthesis (*p* = 9.88E-06), carbon fixation in photosynthetic organisms (*p* = 1.16E-03), biosynthesis of amino acids (*p* = 1.96E-03), carbon metabolism (*p* = 3.25E-02), RNA degradation (*p* = 7.10E-04), and RNA polymerase (*p* = 3.71E-02) (Fig. S7 and Table S11). It is speculated that m^5^C modifications in cyanobacteria may have important regulatory impacts in a variety of processes, including protein translation, metabolic pathways, photosynthesis, and RNA stability.

**Figure 6:**
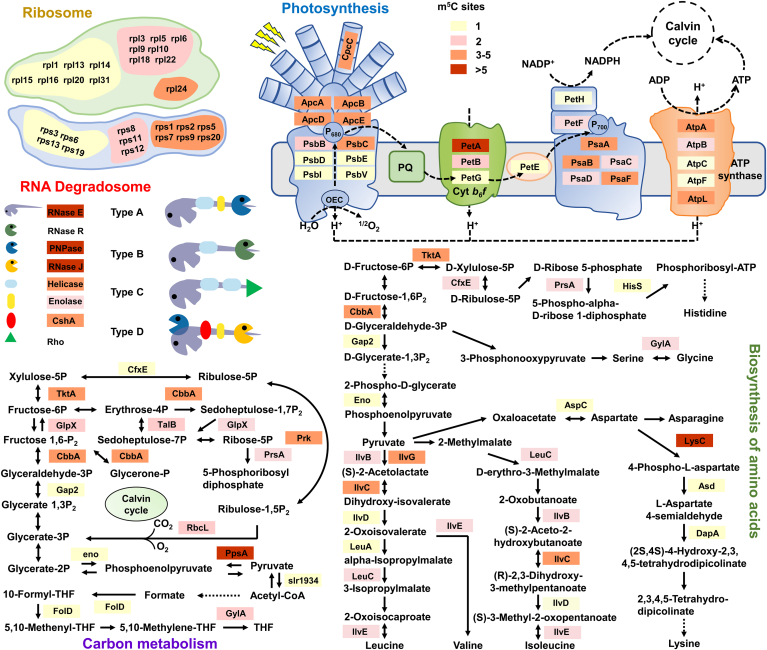
**Schematic illustration of 5-methylcytosine (m^5^C)-modified genes involved in ribosome, RNA degradosome, carbon metabolism, photosynthesis, and biosynthesis of amino acids in *Synechocystis*.** Four distinct colors indicate the number of m^5^C sites per gene: 1, 2, 3 to 5, and >5 sites.

We then mapped the identified m^5^C-modified genes to KEGG pathways to provide a global view of biological pathways. This analysis further highlights the potential regulatory roles of m^5^C in key biological processes, such as photosynthesis and translation. As shown in Figure 6, key enzymes involved in carbon metabolism were also found to be m^5^C modified, such as glycolysis/gluconeogenesis, pentose phosphate pathway, pyruvate metabolism, and Calvin cycle. Since *Synechocystis* is a model organism for photosynthetic studies, we identified 27 genes mapping to photosynthesis. For instance, five genes (*apcA*, *apcB*, *apcD*, *apcE*, and *cpcE*) were found to be resided in phycobilisome, six genes (*psbB*, *psbC*, *psbD*, *psbE*, *psbI*, and *psbV*) were located on photosystem II (PSII), six genes (*petA*, *petB*, *petE*, *PetF*, *petG*, and *petH*) were in cytochrome b^6^f complex, five genes (*psaA*, *psaB*, *psaC*, *psaD*, and *psaF*) were on photosystem I (PSI), and five genes (*atpA*, *atpB*, *atpC*, *atpF*, and *atpL*) were on ATP synthase complex. We anticipated that m^5^C modifications likely modulate photosynthesis and other metabolism by influencing the expression of these genes. Furthermore, we identified 15 ribosomal large subunit genes, of which seven genes contained only one m^5^C site, seven genes contained two m^5^C sites, and *rpl24* possessed more than three m^5^C sites. Among the ribosomal small subunits, four genes had only one m^5^C site, three genes contained two m^5^C sites, and six genes contained more than six m^5^C sites. Notably, we observed eight genes in RNA degradosome, a well-studied complex in bacteria (type A–D). These data suggested that m^5^C may play a pivotal regulatory role in maintaining intracellular protein expression and RNA degradation. Consistent with results of functional annotation, a large subset of identified genes was related to biosynthesis of amino acids, such as *cfxE*, *hisS*, *prsA*, and *tktA* genes in histidine biosynthesis; *gylA* in glycine biosynthesis; *cbba*, *gap2*, *eno*, *iivB*, *iivC*, *iiVD*, *iivE*, *leuA*, and *leuC* in leucine, isoleucine, and valine synthesis; and *lysC*, *asd*, *dapA*, and *aspC* in lysine and asparagine synthesis. Consequently, the m^5^C RNA methylation may be a likely mechanism responsible for the regulation of protein expression, RNA degradation, photosynthesis, and metabolism.

### m^5^C modification negatively affects translation efficiency in *Synechocystis*

**Figure 7:**
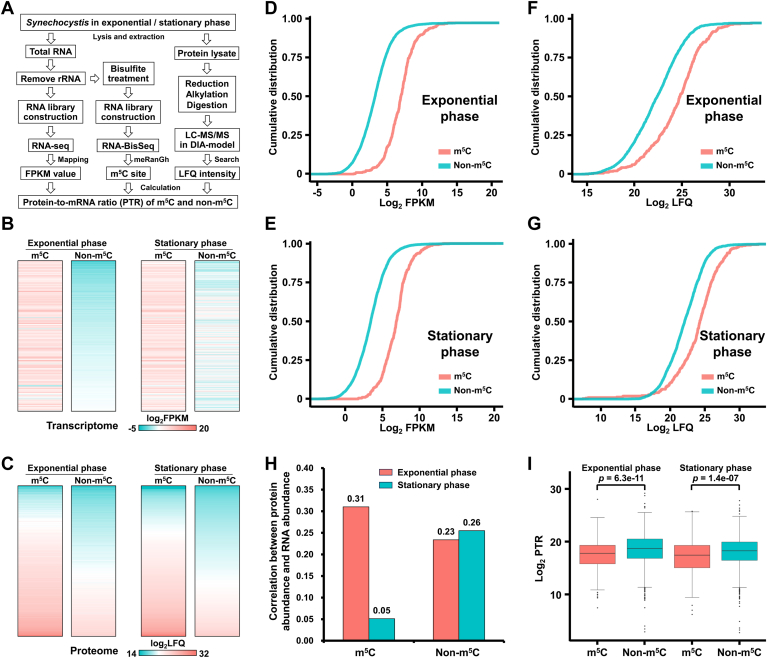
**5-Methylcytosine (m^5^C) modification contributes to protein abundance in *Synechocystis*.***A,* integrated workflow combining transcriptomic, proteomic, and m^5^C methylome analysis of *Synechocystis* from exponential and stationary growth phases. *B,* heatmap showing RNA and protein abundance of genes with and without m^5^C modification in *Synechocystis* from both exponential and stationary growth phases. The log_2_ values of LFQ and RPKM abundance are plotted. *C,* heatmap showing protein abundance of genes with and without m^5^C modification in *Synechocystis* from both exponential and stationary growth phases. The log_2_ values of LFQ abundance are plotted. *D* and *E,* cumulative distribution of mRNA expression levels between genes with and without m^5^C in *Synechocystis* from exponential (*D*) and stationary (*E*) phases. *F* and *G,* cumulative distribution of protein expression levels between genes with and without m^5^C in *Synechocystis* from the exponential phase (*F*) and the stationary phase (*G*). *H,* histogram depicting the Spearman’s correlation between RNA and protein abundance in genes with and without m^5^C in *Synechocystis* from both exponential and stationary growth phases. The Spearman’s rank correlations were calculated between log_2_ FPKM (transcriptomics) and log_2_ LFQ (proteomics) values of genes with or without m^5^C modification. *I,* boxplot comparison of the protein-to-RNA ratio (PTR) between genes with and without m^5^C in *Synechocystis* from both exponential and stationary growth phases. Statistical significance was determined using the Wilcoxon rank sum test. FPKM, fragments per kilobase of transcript per million mapped read; LFQ, label-free quantitation; RPKM, reads per kilobase of transcript per million mapped read.

To explore the impact of m^5^C modification on protein expression in *Synechocystis*, we performed integrated transcriptomic, proteomic, and m^5^C methylome analyses from the same *Synechocystis* cultures grown in exponential phase and stationary phase (Fig. 7*A*). In total, we identified 3601 genes from the exponential phase and 3635 genes from the stationary phase at the transcriptomic level (Fig. S8, *A* and *B* and Table S12); 2205 proteins from the exponential phase and 2113 proteins from the stationary phase at the proteomic level (Fig. S8 *A* and *B* and Table S13). Although 2184 genes (60.3%) from the exponential phase (60.3%) and 2098 genes from the stationary phase (57.5%) were detected in both datasets, a small fraction of proteins from the exponential phase (21, 0.6%) and proteins from the stationary phase (15, 0.4%) identified in the proteomic datasets lacked corresponding transcripts (Fig. S8 *A* and *B*). To evaluate the reproducibility of our transcriptomic and proteomic results, we conducted correlation analysis of the mRNA abundance and protein abundance using the log_2_-transformed FPKM values and label-free quantification intensities. The Spearman’s correlation coefficients were all greater than 0.92, indicating high repeatability across the three biological replicates (Fig. S8, *C*–*F*). The heatmaps of mRNA abundance (log_2_ FPKM) and protein abundance (log_2_LFQ) revealed that genes with m^5^C exhibited significantly higher expression levels than genes without m^5^C (Fig. 7, *B* and *C* and Table S12–13). This was further supported by cumulative distribution analyses, which showed that genes with m^5^C have higher expression levels at both mRNA and protein levels compared with genes without m^5^C (Fig. 7, *D*–*G*). Our quantitative transcriptomic, proteomic, and m^5^C methylome derived from the same *Synechocystis* cultures enable us to elucidate the role of m^5^C modification in regulating protein levels. We found that genes with m^5^C exhibited significantly higher correlation coefficients in the exponential phase, and significantly lower correlation coefficients in the stationary phase, than those without m^5^C (*p* < 0.001) (Fig. 7*H*), implying that m^5^C plays an important role in determining the mRNA–protein discordance. To further assess the effect of m^5^C modification on translational efficiency, we calculated the PTR to evaluate the variation of protein and RNA levels as described previously (43). Overall, we found a significantly lower PTR for genes with m^5^C than for genes without m^5^C during exponential and stationary phases, indicating a translation-repressive role of m^5^C in *Synechocystis* (Fig. 7*I* and Table S14*A*). However, not every mRNA with m^5^C exhibits reduced protein expression, compared with non–m^5^C-modified genes. According to our PTR analysis**,** we calculated the median PTR values for m^5^C-modified and non–m^5^C-modified genes. Notably, 116 m^5^C-modified genes exhibited PTR values above the median PTR value of non–m^5^C-modified genes in the exponential phase, and the PTR values of 142 m^5^C-modified genes exceeded the median PTR value of non–m^5^C-modified genes in the stationary phase (Table S14*B*). Together, our findings suggested that m^5^C modification may exert a negative impact on protein abundance and play a role in the post-transcriptional regulation of mRNA, similar to the regulatory mechanisms of m^5^C observed in other species (52, 53).

## Discussion

To the best of our knowledge, this is the first systematic study of RNA modifications in cyanobacteria. In this study, we identified 21 different RNA modifications and obtained the transcriptome-wide landscape of m^5^C modification in a model cyanobacterium. Our m^5^C methylome data indicated that m^5^C-modified genes were involved in diverse cellular processes in *Synechocystis*, including protein translation, metabolic pathways, and photosynthesis. In processes, such as light harvesting, PSI and PSII, we found that representative genes from the photosynthetic pathway harbored m^5^C modification. Thus, understanding the physiological functions of m^5^C modification on these genes would certainly help to elucidate the post-transcriptional regulation of photosynthesis in cyanobacteria, as well as in other photosynthetic organisms.

Using an MRM-based MS strategy, we identified and quantified 21 different RNA modifications of *Synechocystis*, revealing a distinct utilization pattern of RNA modification in cyanobacteria compared with eukaryotes. The highly abundant m^6^A modification observed in eukaryotes is notably scarce in cyanobacteria, constituting a mere 0.005% of total adenine modifications, equivalent to a mere one-tenth of the proportion found in eukaryotes (54, 55, 56). Meanwhile, we have found that cyanobacteria prefer uridine-type RNA modifications, with U-type modifications such as m^5^U and ψ exceeding 2% in uridines. In addition, extensive RNA modifications have been observed to be regulated in response to different stress conditions (Fig. 3). This phenomenon aligns with previous studies demonstrating that RNA modifications play central roles in stress response across various species. For example, *Synechocystis* exhibits elevated m^6^A levels under heat stress, mirroring observations in *Drosophila*, where exposure to heat stress leads to increased Mettl3-dependent m^6^A modifications in the brain, thereby dampening neural stress responses (57). Under oxidative stress induced by reactive oxygen species, m^6^A modification may mediate the inhibition of the KEAP1 pathway and activation of the NRF2 pathway, thereby regulating colistin-induced oxidative stress (58, 59). Similarly, in *A. thaliana*, HL stress alters m^6^A modification levels of numerous photosynthesis-related transcripts. This regulation depends on the methyltransferase VIRILIZER, which mediates m^6^A modification of transcripts encoding photoprotective proteins and their regulators, maintaining RNA stability and translation efficiency to preserve photosynthetic capacity under intense light (60). In contrast, pseudouridine levels decrease under cold stress (Fig. 3), a trend also reported in rice, where the pseudouridine synthase OsPUS1 accumulates and catalyzes pseudouridylation of chloroplast precursor rRNAs to facilitate pre-rRNA processing, thereby contributing to cold tolerance (61). Moreover, the reduction of m^5^C on tRNAs has been shown to cause translation stalling, triggering cells exposed to oxidative stress to enter a catabolic state (62). Our global nucleoside measurements detected significant changes in total m^5^C only when comparing HL to HT conditions (Fig. 3), whereas other stress treatments did not show statistically significant differences relative to normal growth. However, the m^5^C abundance measured by LC–MS/MS represents the sum of all modified cytosines across all RNA species. It is therefore possible that specific transcripts or cytosine positions undergo dynamic methylation changes in response to stress, even if the global m^5^C level appears unchanged. For example, in *T. kodakarensis*, although 27 nucleoside modifications were identified in total RNA, 17 modifications remained constant across temperature changes, including m^5^C, m^7^G (63). Yet, Bis-Seq revealed that both the number and distribution of m^5^C sites, as well as the set of modified transcripts, varied between exponential and stationary growth phases, indicating a dynamic m^5^C landscape responsive to environmental cues (14). Consistent with this, our study found that while we did not directly compare single-nucleotide m^5^C levels under stress, the correlation between transcript and protein abundance was higher for m^5^C-modified genes than for unmodified ones during exponential growth. In contrast, during the stationary phase, this correlation markedly decreased for m^5^C-modified genes. This shift suggests that m^5^C exerts a stronger influence on translation efficiency during stationary growth, likely mediated by dynamic changes in m^5^C stoichiometry, consistent with previous findings of m^5^C epitranscriptomic in *T. kodakarensis* (14) and *E. coli* (64). Overall, our results reveal notable stress-induced alterations in different RNA modifications in *Synechocystis* (Fig. 3), underscoring their potential biological significance. The MRM-based quantitative framework established here offers a powerful tool for monitoring RNA modification dynamics over time and under diverse stress conditions. Importantly, this approach can be readily adapted to investigate RNA modifications in other sequenced prokaryotic organisms.

The m^5^C modification has been found to play important roles in maintaining RNA metabolism, responding to stress, and cell proliferation in *Arabidopsis* and other eukaryotic cells (65, 66). However, the specific sites, functions, and regulatory mechanisms of m^5^C modification remain largely unknown in cyanobacteria. Here, we compiled the first m^5^C methylome in cyanobacteria using a Bis-Seq strategy, providing a valuable resource for future studies of the regulation and functions of this important RNA modification. This strategy has enabled the identification of m^5^C RNA modifications in various species. For instance, in *Drosophila melanogaster*, 8974 m^5^C sites were identified on exonic regions, and embryos lacking maternal mRNA m^5^C display delayed cell cycle and fail to timely initiate maternal-to-zygotic transition (67). In *A. thaliana*, researchers developed m^5^C RNA maps for mRNAs and noncoding RNAs and identified over 1000 m^5^C sites in three tissue types, highlighting the role of m^5^C modification in regulating root development and enhancing sensitivity to oxidative stress (2). In this study, we identified 824 m^5^C methylation sites in the mRNA of 382 genes (Table S5), surpassing 10% of the total gene count in *Synechocystis*. Further validation by m^5^C-RIP confirmed 331 overlapping sites (40.17%) distributed across 145 genes and 129 peaks, underscoring the high quality and reliability of our methylome data. Notably, recent studies in *T. kodakarensis* reported a similar pattern, with approximately ∼10% of all transcripts containing m^5^C (14). Interestingly, five genes contained more than 10 m^5^C modification sites, with the gene *pnp* harboring a remarkable 17 m^5^C sites. Motif analysis revealed that m^5^C modifications tend to localize in a G-rich environment in cyanobacteria (Fig. 4*C*), a pattern slightly different from that reported in other organisms (47). This result suggests the presence of unique reader proteins in cyanobacteria that recognize m^5^C modifications and exert distinct biological functions. Through further functional analysis, we found that m^5^C modifications may regulate protein translation, photosynthesis, and carbon metabolic processes in cyanobacteria (Fig. 6). Specifically, 27 photosynthesis-related proteins were found to harbor m^5^C modifications, primarily localized in the phycobilisome, PSI, PSII, the cytochrome b_6_f complex, and ATP synthase (Fig. 6). For example, transcripts encoding *petA*, *psaA*, and *apcB*, core components of photosynthetic complexes, carry m^5^C sites, forming an m^5^C-methylated interaction network implicated in photosynthetic regulation (Fig. S6). These findings suggest that m^5^C may influence the stability or translation efficiency of these transcripts, thereby modulating photosynthetic flux. Similar regulatory roles of RNA methylation in photosynthesis have been documented in plants. In rice, the m^5^C methyltransferase OsNSUN2 methylates chloroplast transcripts, and its loss impairs photosynthetic capacity (68). In *Arabidopsis*, changes in TRM4B-mediated m^5^C levels alter chloroplast gene expression and reduce photosynthetic efficiency (66). Likewise, the m^6^A methyltransferase VIRILIZER regulates transcripts encoding photoprotective proteins, preserving their stability and translation efficiency under HL stress (60). Although no previous studies have investigated cyanobacterial photosynthesis from the perspective of m^5^C modification, these precedents support the plausibility of a similar post-transcriptional regulatory mechanism in cyanobacteria. Given the prevalence of m^5^C on photosynthetic genes, elucidating its physiological roles will be essential for understanding RNA-based regulation of photosynthesis in cyanobacteria as well as in plants.

Through integrative analyses combining transcriptomics, proteomics, and m^5^C methylomics, we found that m^5^C modifications generally enhance gene expression and increase protein abundance (Fig. 7). Similarly, in *Helicobacter pylori*, deletion of the DNA m^5^C methyltransferase M.Hpy99III significantly reduces expression of a reporter gene (69). In human oral squamous cell carcinoma cells, loss of m^5^C at position 34 of mitochondrial tRNA^Met^ downregulates expression of the oxidative phosphorylation complex proteins (70). However, we also observed instances where m^5^C led to discrepancies between RNA and protein levels (Fig. 7*H*), suggesting that the regulatory effects of m^5^C are context dependent. Indeed, under different growth conditions, *Synechocystis* displayed varying m^5^C effects on RNA–protein correspondence. Similar context-dependent regulation has been reported in mammalian cells. Under lactic acid stress, enhanced binding of NSUN2 to m^5^C increases the expression of ENO1 and MEK1 *via* m^5^C-mediated stabilization (71). In pumpkin, deletion of both m^5^C and m^6^A modification sites from the CmoCK1 mRNA abolishes its mobility and prevents proper translation, disrupting protein expression (72). Our findings that m^5^C-modified genes exhibit significantly lower PTRs than unmodified genes (Fig. 7*I*) suggest that m^5^C may function as a translational repressor in *Synechocystis*, consistent with observations in other species (52, 53). In mammals, m^5^C sites are often enriched near translation initiation regions, a distribution pattern conserved between humans and mice (73). In mouse neural stem cells, polysome profiling suggests that m^5^C modification may impact mRNA translation efficiency (74), whereas in human HeLa cells, steric hindrance from m^5^C reader proteins likely interferes with ribosome progression, leading to translational repression (75). Beyond mRNA regulation, m^5^C modifications in rRNAs also contribute to translational control. In yeast, m^5^C2278 in 25S rRNA maintains ribosomal structure during oxidative stress, aiding selective mRNA recruitment and translation in cellular signal responses (76). Conversely, loss of NSUN5-mediated m^5^C3782 in 28S rRNA impairs global protein synthesis (77, 78). These studies collectively support a model in which m^5^C serves as a versatile regulator of translation and stress adaptation across domains of life. Taken together, our findings reveal that m^5^C modification plays a key role in fine-tuning translation efficiency, photosynthetic activity, and stress responses in cyanobacteria. A more detailed underlying molecular mechanism would necessitate the identification of m^5^C reader(s) and eraser(s) with specificity for targeting specific modification sites.

In summary, we provide a holistic view of RNA modifications and report the first RNA m^5^C methylome in cyanobacteria, which present a critical database for functional analyses of RNA modifications in cyanobacteria. Our integrated multiomics analyses elucidate the role of m^5^C modification in affecting protein abundance and provide insights into the post-transcriptional regulation of photosynthesis in cyanobacteria. We expect that the methods used in this study will be widely applicable to any sequenced prokaryotic organism, which will yield similar or greater sets of novel discoveries.

### Statistical analysis

Data are presented as means ± SD from at least three independent experiments. Statistical analyses were conducted using the R framework (version 4.3.1) or Excel, employing two-sample *t* tests, ANOVA, or the Wilcoxon rank sum test. A *p* value of <0.05 was considered statistically significant.

## Data availability

The raw MS data have been deposited in the public access iprox database (http://www.iprox.org) with the identifier IPX0009594000. The raw Bis-Seq and RNA-Seq data have been deposited in the National Center for Biotechnology Information, under accession numbers PRJNA1165008 (https://www.ncbi.nlm.nih.gov/bioproject/?term=PRJNA1165008) and PRJNA1275829 (https://www.ncbi.nlm.nih.gov/bioproject/?term=PRJNA1275829).

## Supporting information

This article contains supporting information.

## Conflict of interest

The authors declare that they have no conflicts of interest with the contents of this article.
