# Supplementary material for: Global discovery of RNA modifications and functional analysis of m5C methylome in cyanobacteria
Source: J Biol Chem. 2026 Jan 7;302(2):111133. doi: 10.1016/j.jbc.2026.111133 (PMC12860351; doi:10.1016/j.jbc.2026.111133)
Supplement: Table S3 [file mmc3.docx]

**Table S3**

**Unmodified_Control_RNA**

GGAGACCCAAGCTTGGTACCGAGCTCGGATCCGCCACCATGAAGACCTTAATTCTTGCCGTTGCATTAGTCTACTGCGCCACTGTTCATTGCCAGGACTGTCCTTACGAACCTGATCCACCAAACACAGTTCCAACTTCCTGTGAAGCTAAAGAAGGAGAATGTATTGATAGCAGCTGTGGCACCTGCACGAGAGACATACTATCAGATGGACTGTGTGAAAATAAACCAGGAAAAACATGTTGCCGAATGTGTCAGTATGTAATTGAATGCAGAGTAGAGGCCGCAGGATGGTTTAGAACATTCTATGGAAAGAGATTCCAGTTCCAGGAACCTGGTACATACGTGTTGGGTCAAGGAACCAAGGGCGGCGACTGGAAGGTGTCCATCACCCTGGAGAACCTGGATGGAACCAAGGGGGCTGTGCTGACCAAGACAAGACTGGAAGTGGCTGGAGACATCATTGACATCGCTCAAGCTACTGAGAATCCCATCACTGTAAACGGTGGAGCTGACCCTATCATCGCCAACCCGTACACCATCGGCGAGGTCACCATCGCTGTTGTTGAGATGCCAGGCTTCAACATCACCGTCATTGAGTTCTTCAAACTGATCGTGATCGACATCCTCGGAGGAAGATCTGTAAGAATCGCCCCAGACACAGCAAACAAAGGAATGATCTCTGGCCTCTGTGGAGATCTTAAAATGATGGAAGATACAGACTTCACTTCAGATCCAGAACAACTCGCTAATCAGCCTAAGATCAACCAGGAGTTTGACGGTTGTCCACTCTATGGAAATCCTGATGACGTTGCATACTGCAAAGGTCTTCTGGAGCCGTACAAGGACAGCTGCCGCAACCCCATCAACTTCTACTACTACACCATCTCCTGCGCCTTCGCCCGCTGTATGGGTGGAGACGAGCGAGCCTCACACGTGCTGCTTGACTACAGGGAGACGTGCGCTGCTCCCGAAACTAGAGGAACCTGCGTTTTGTCTGGACATACTTTCTACGATACATTTGACAAAGCAAGATACCAATTCCAGGGTCCCTGCAAGGAGATTCTTATGGCCGCCGACTGTTTCTGGAACACTTGGGATGTGAAGGTTTCACACAGGAATGTTGACTCTTACACTGAAGTAGAGAAAGTACGAATCAGGAAACAATCGACTGTAGTAGAACTCATTGTTGATGGAAAACAGATTCTGGTTGGAGGAGAAGCCGTGTCCGTCCCGTACAGCTCTCAGAACACTTCCATCTACTGGCAAGATGGTGACATACTGACTACAGCCATCCTACCTGAAGCTCTGGTGGTCAAGTTCAACTTCAAGCAACTGCTCGTCGTACATATTAGAGATCCATTCGATGGTAAGACTTGCGGTATTTGCGGTAACTACAACCAGGATTTCAGTGATGATTCTTTTGATGCTGAAGGAGCCTGTGATCTGACCCCCAACCCACCGGGATGCACCGAAGAACAGAAACCTGAAGCTGAACGACTCTGCAATAGTCTCTTCGCCGGTCAAAGTGATCTTGATCAGAAATGTAACGTGTGCCACAAGCCTGACCGTGTCGAACGATGCATGTACGAGTATTGCCTGAGGGGACAACAGGGTTTCTGTGACCACGCATGGGAGTTCAAGAAAGAATGCTACATAAAGCATGGAGACACCCTAGAAGTACCAGATGAATGCAAATAGGCGGCC
