# Supplementary material for: Global discovery of RNA modifications and functional analysis of m5C methylome in cyanobacteria
Source: J Biol Chem. 2026 Jan 7;302(2):111133. doi: 10.1016/j.jbc.2026.111133 (PMC12860351; doi:10.1016/j.jbc.2026.111133)
Supplement: Supplementary Figures [file mmc15.docx]

**Supplementary Figures**


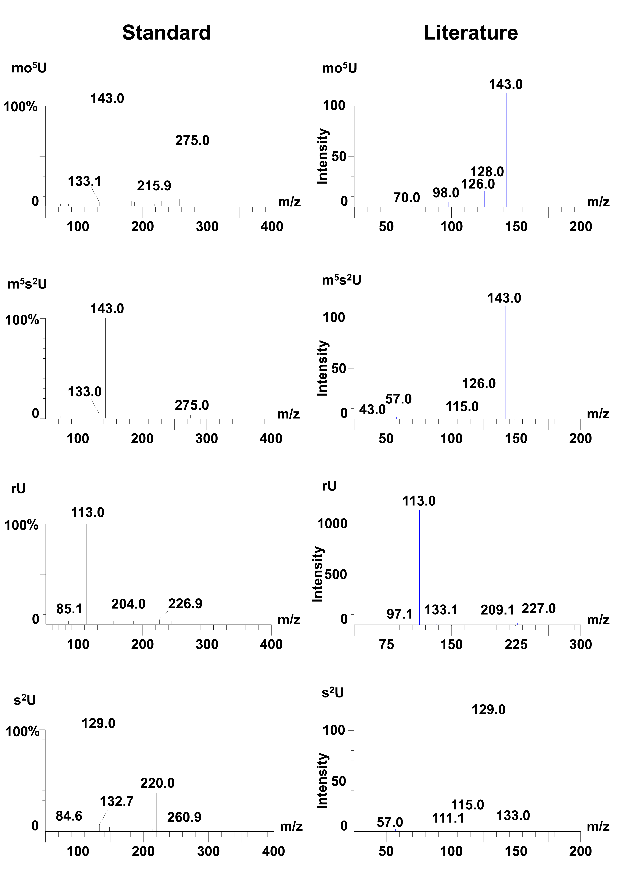

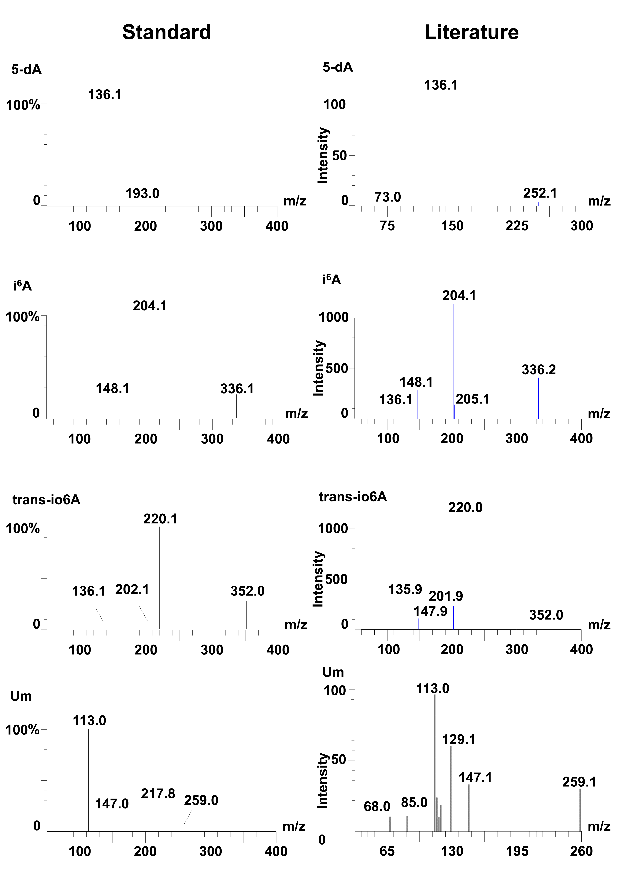


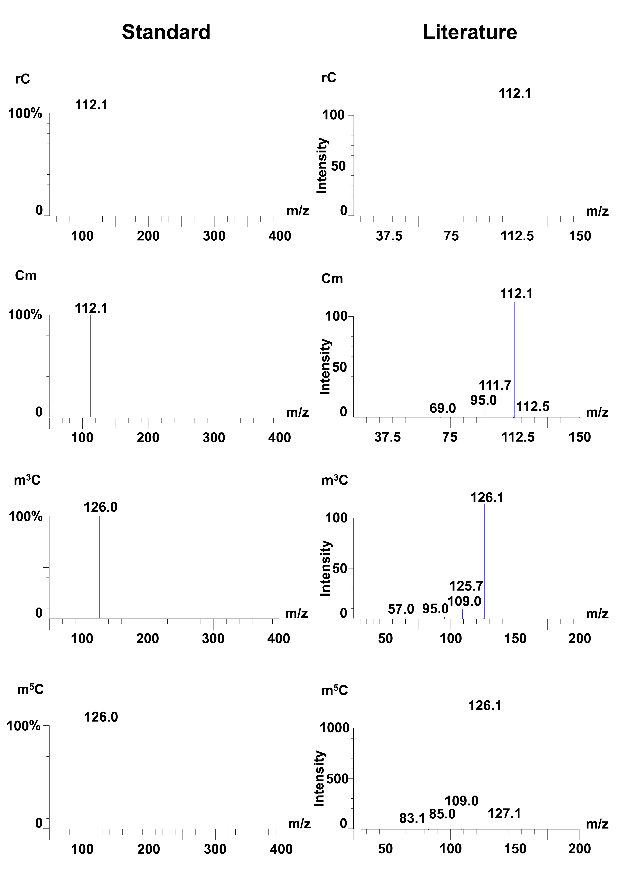

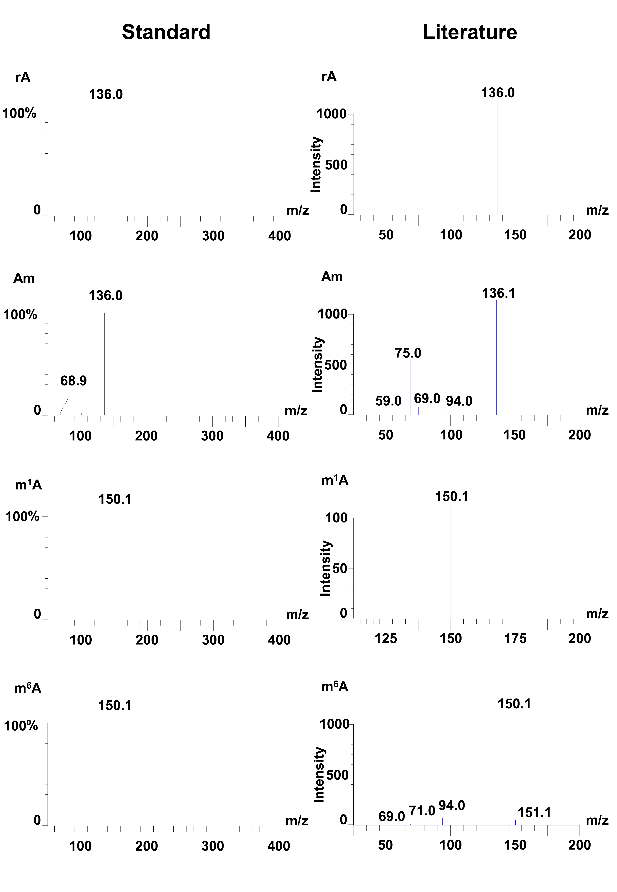


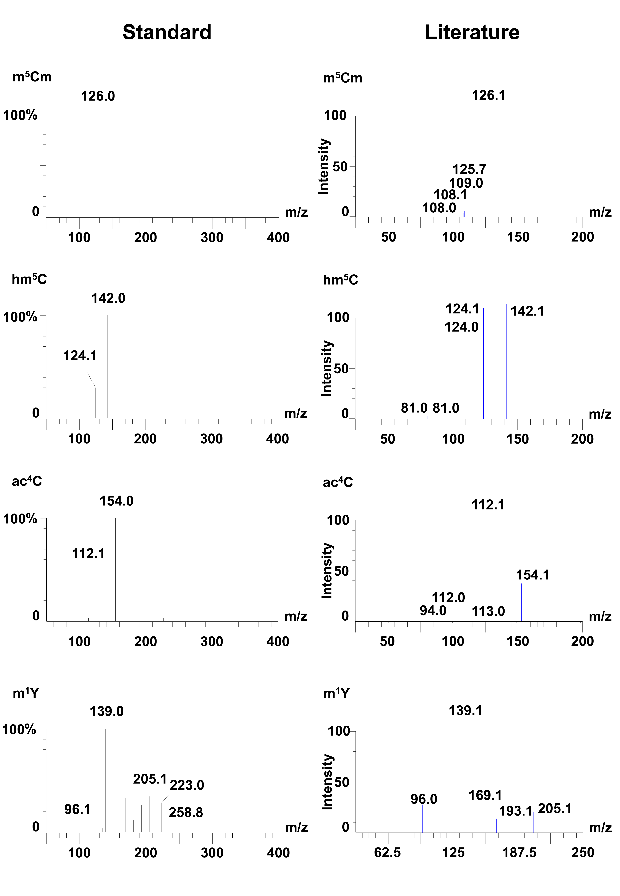

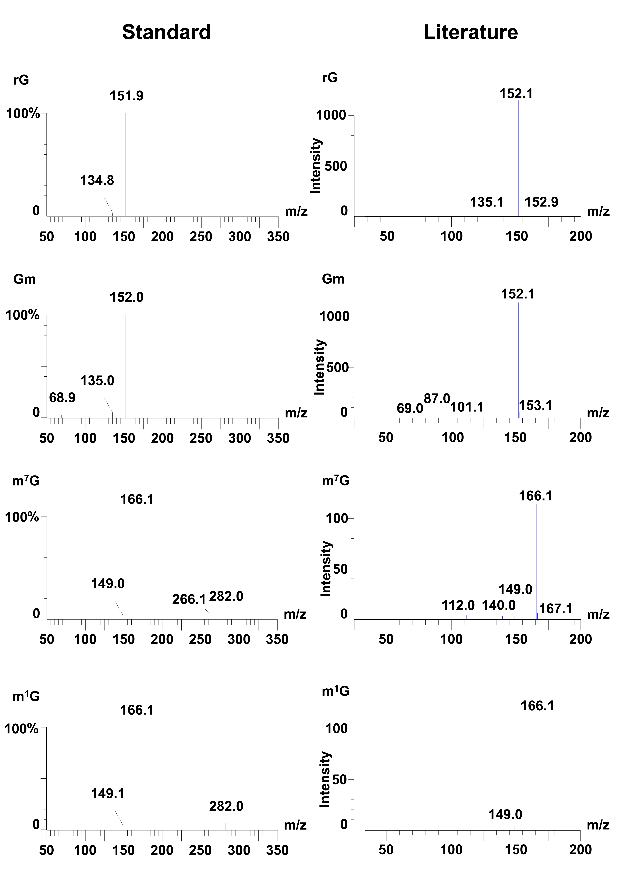


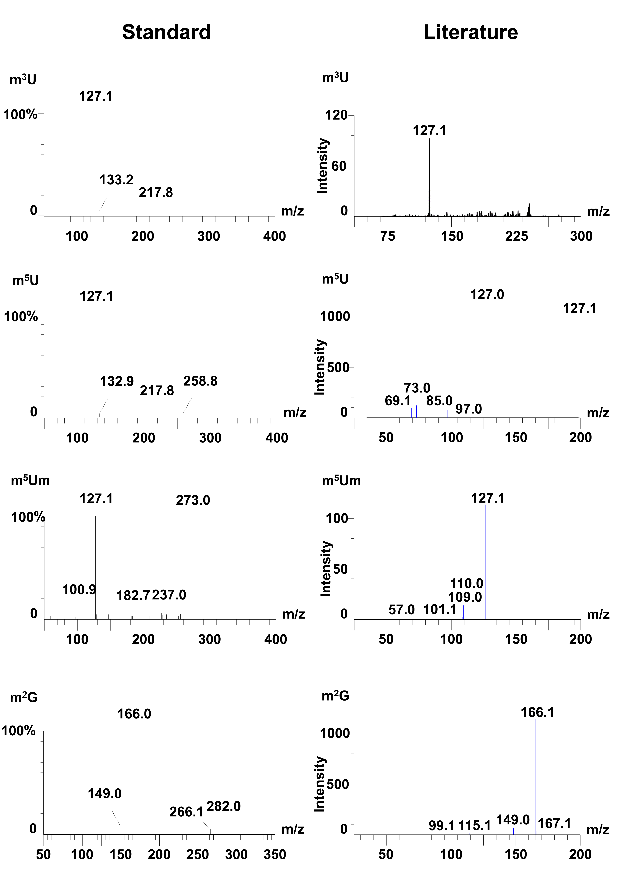

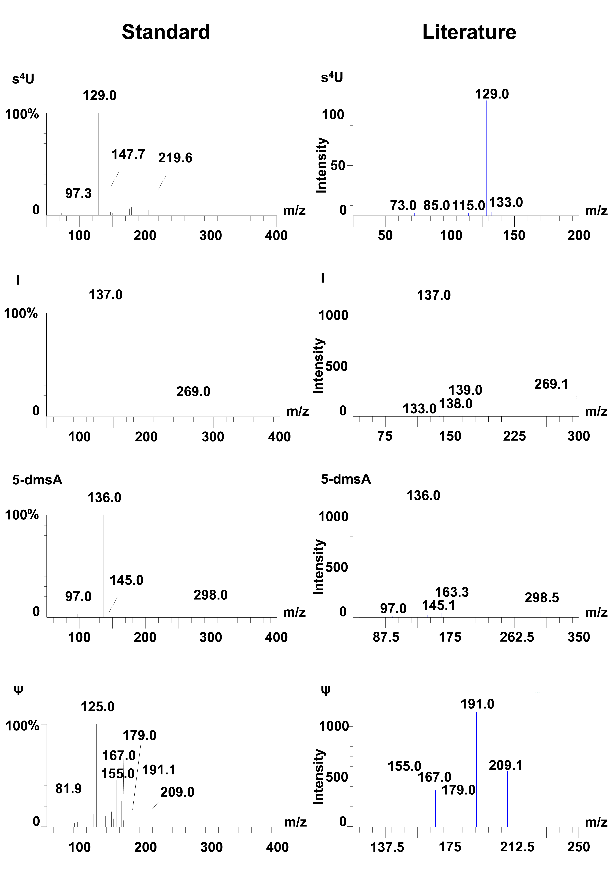


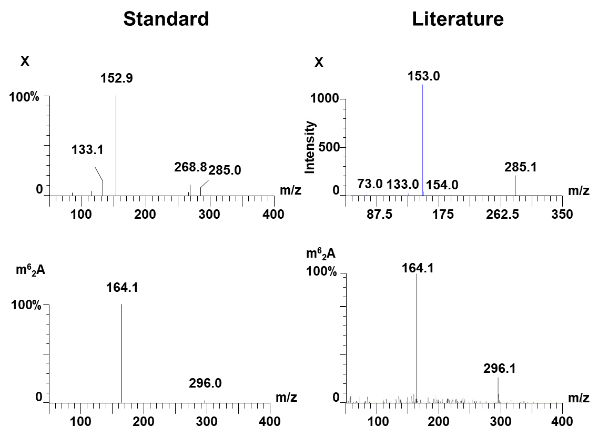

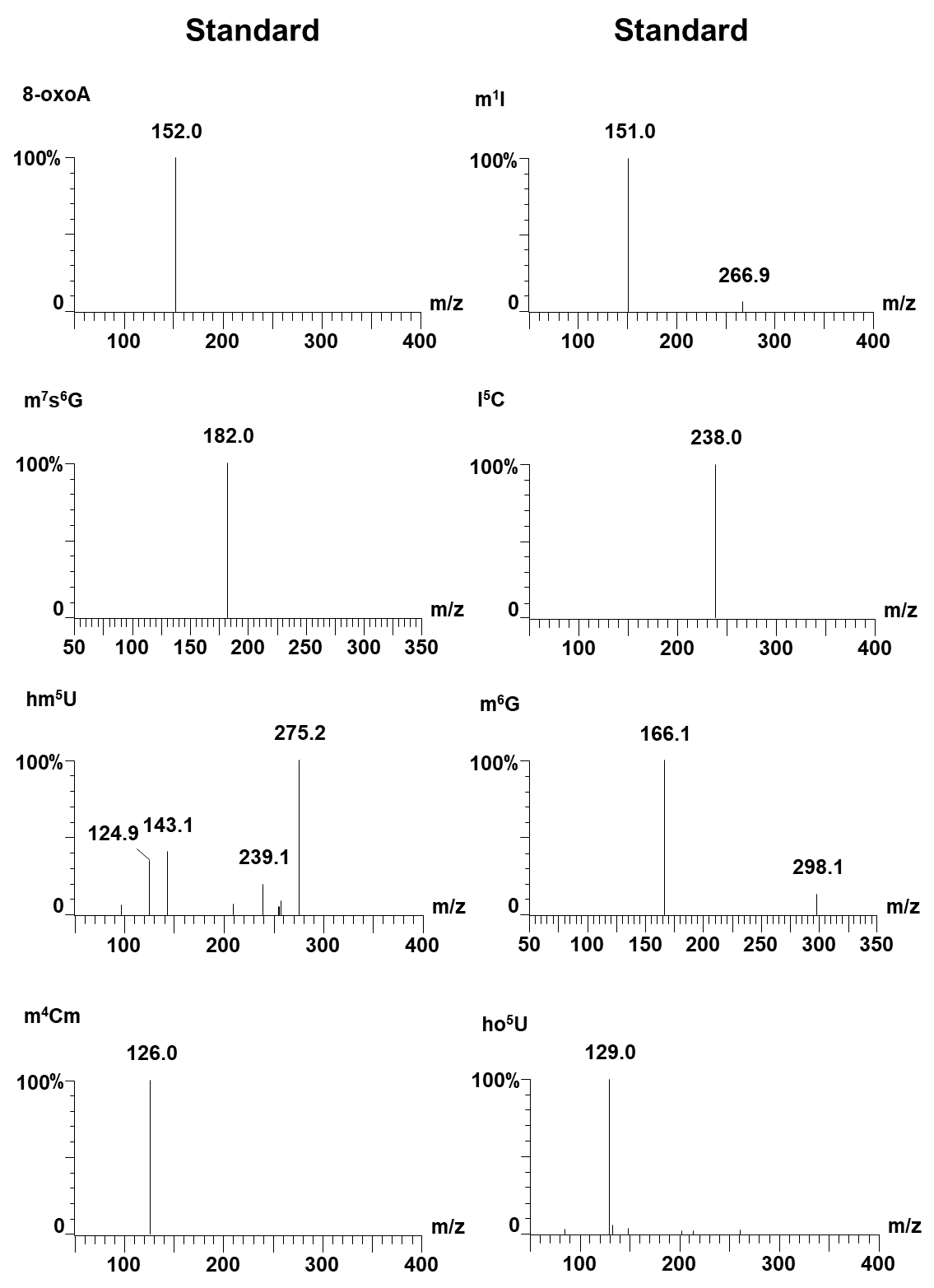


**Figure S1**. Veriﬁcation of nucleosides by comparing the tandem MS/MS spectra from the synthesized 42 nucleoside standards with those obtained on PubChem.


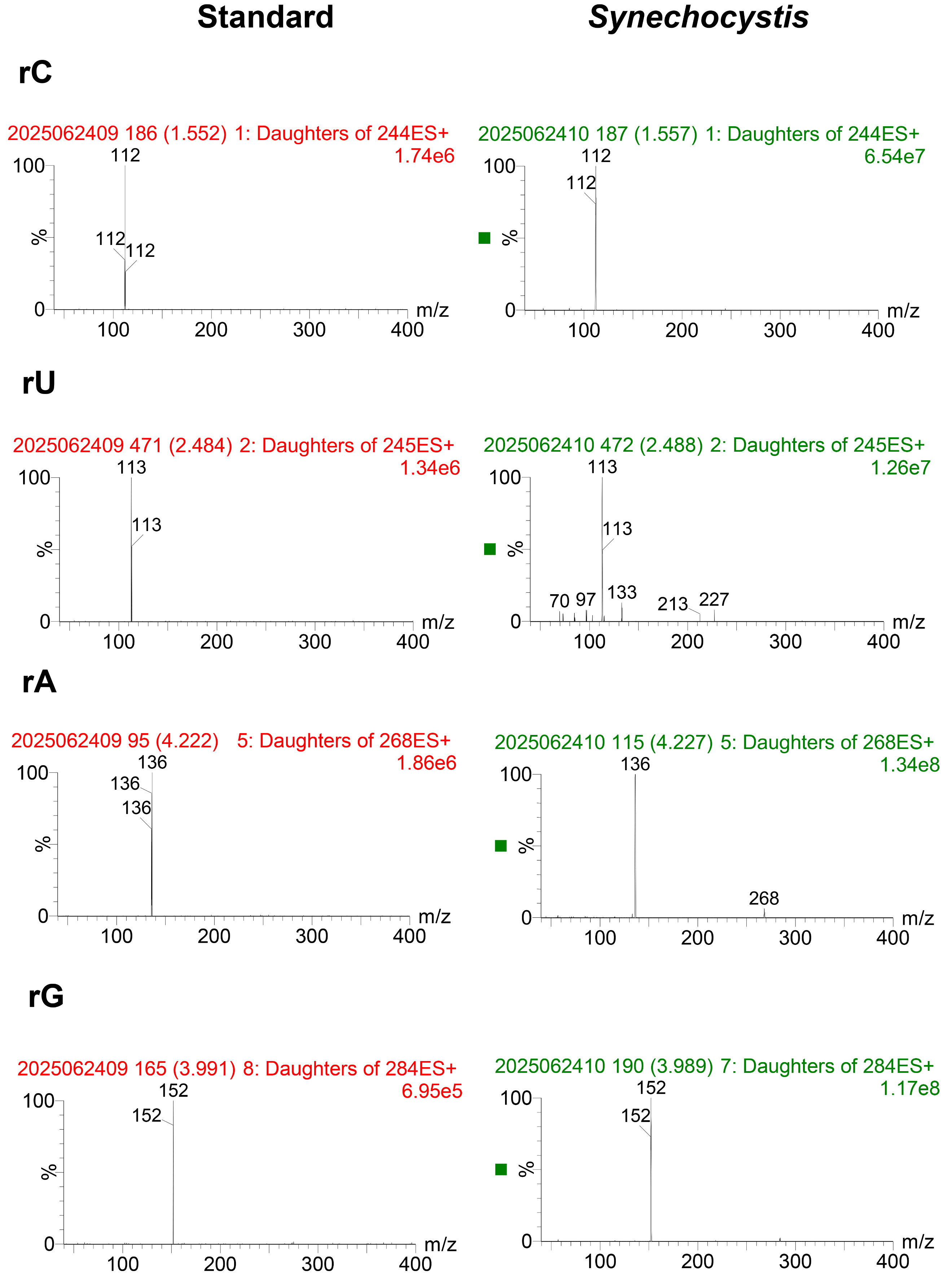

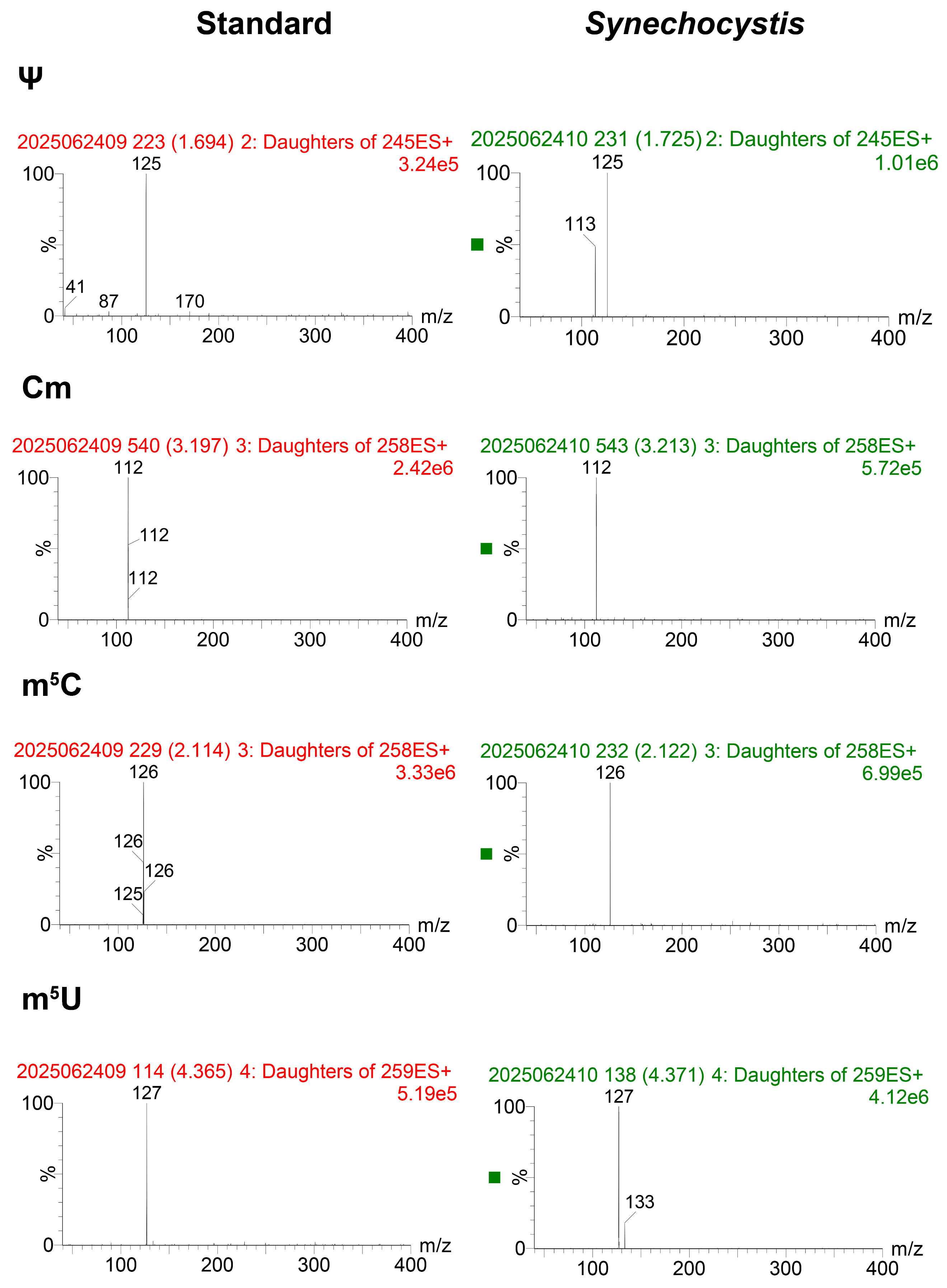

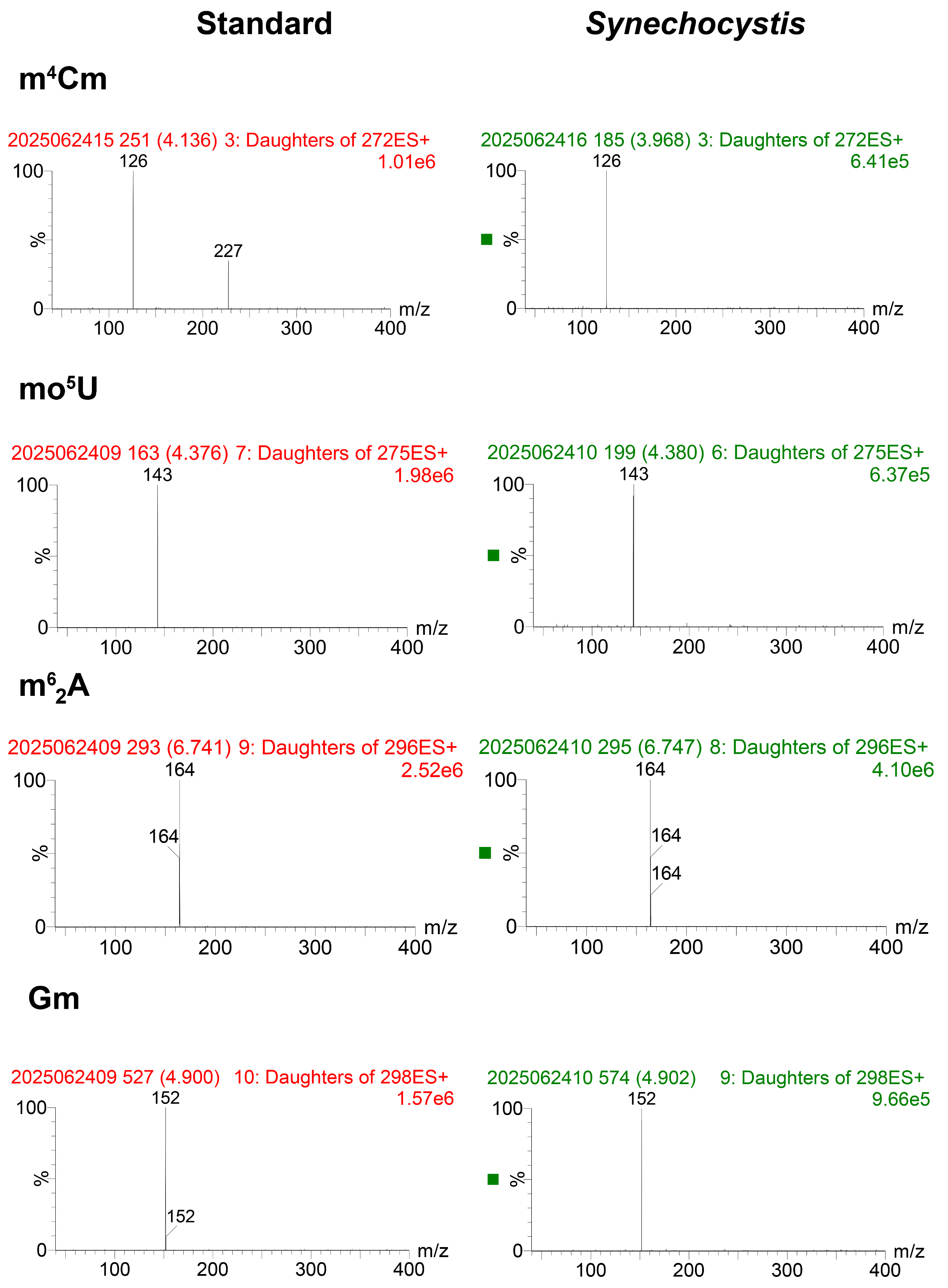

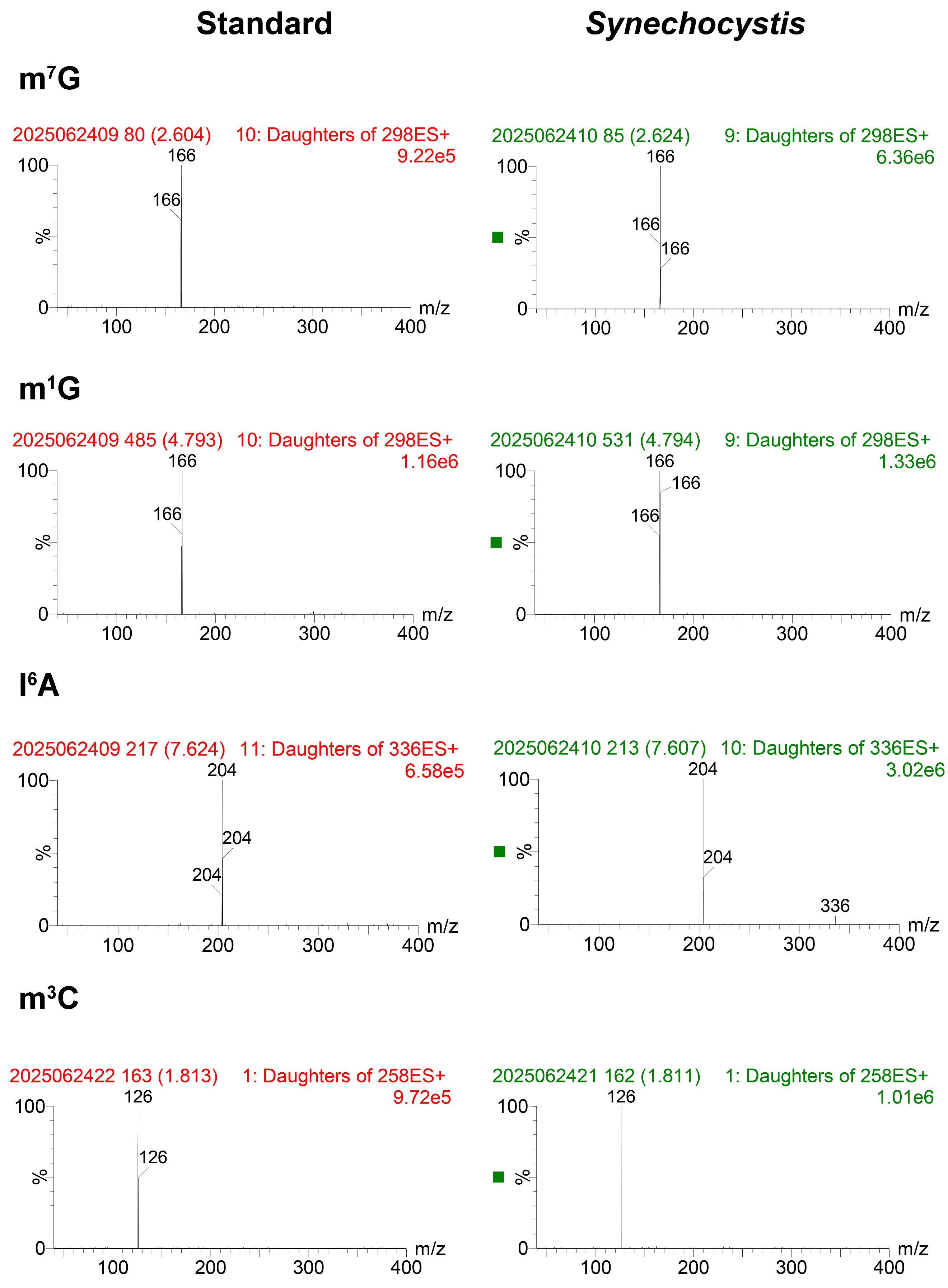


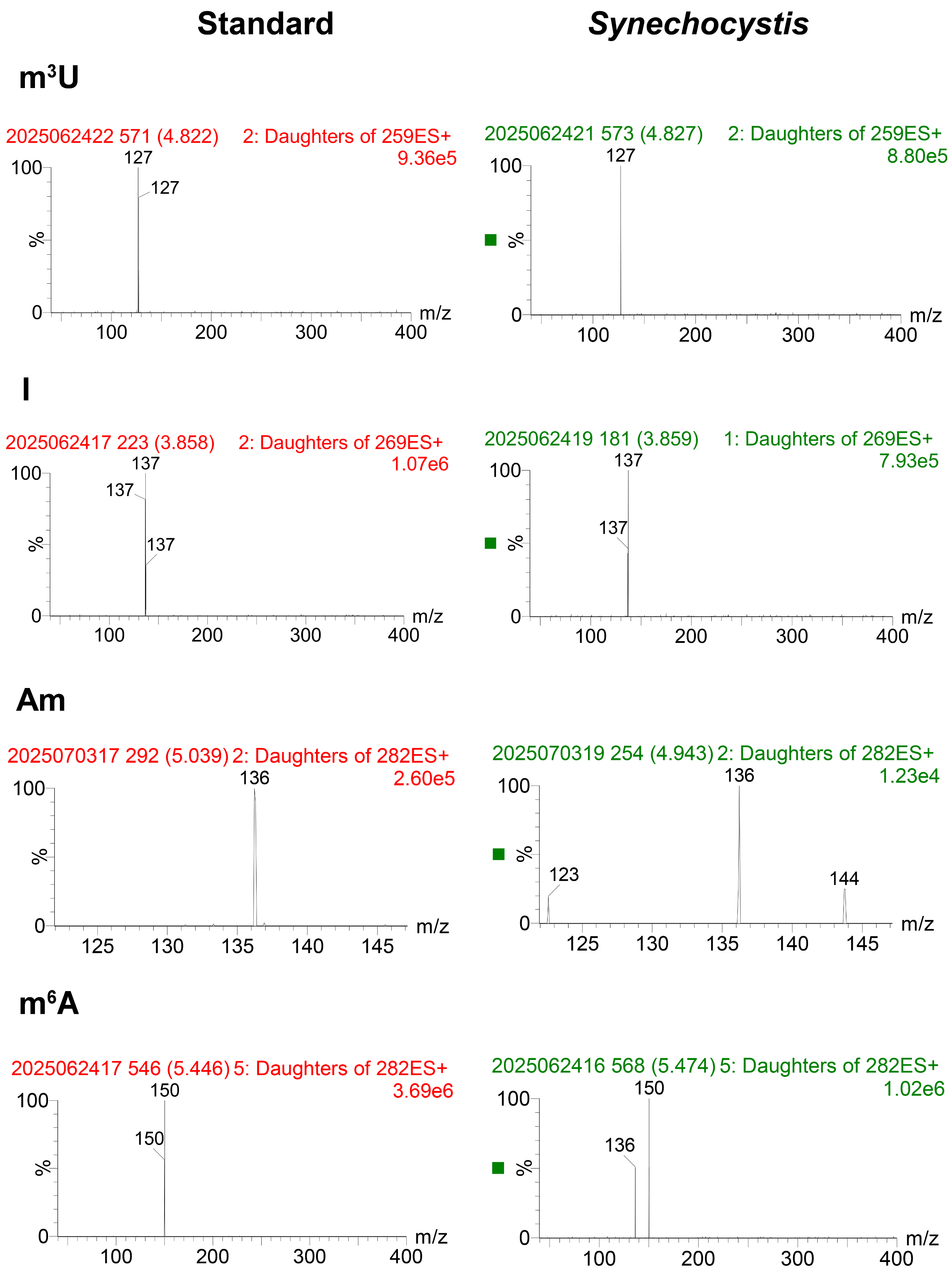

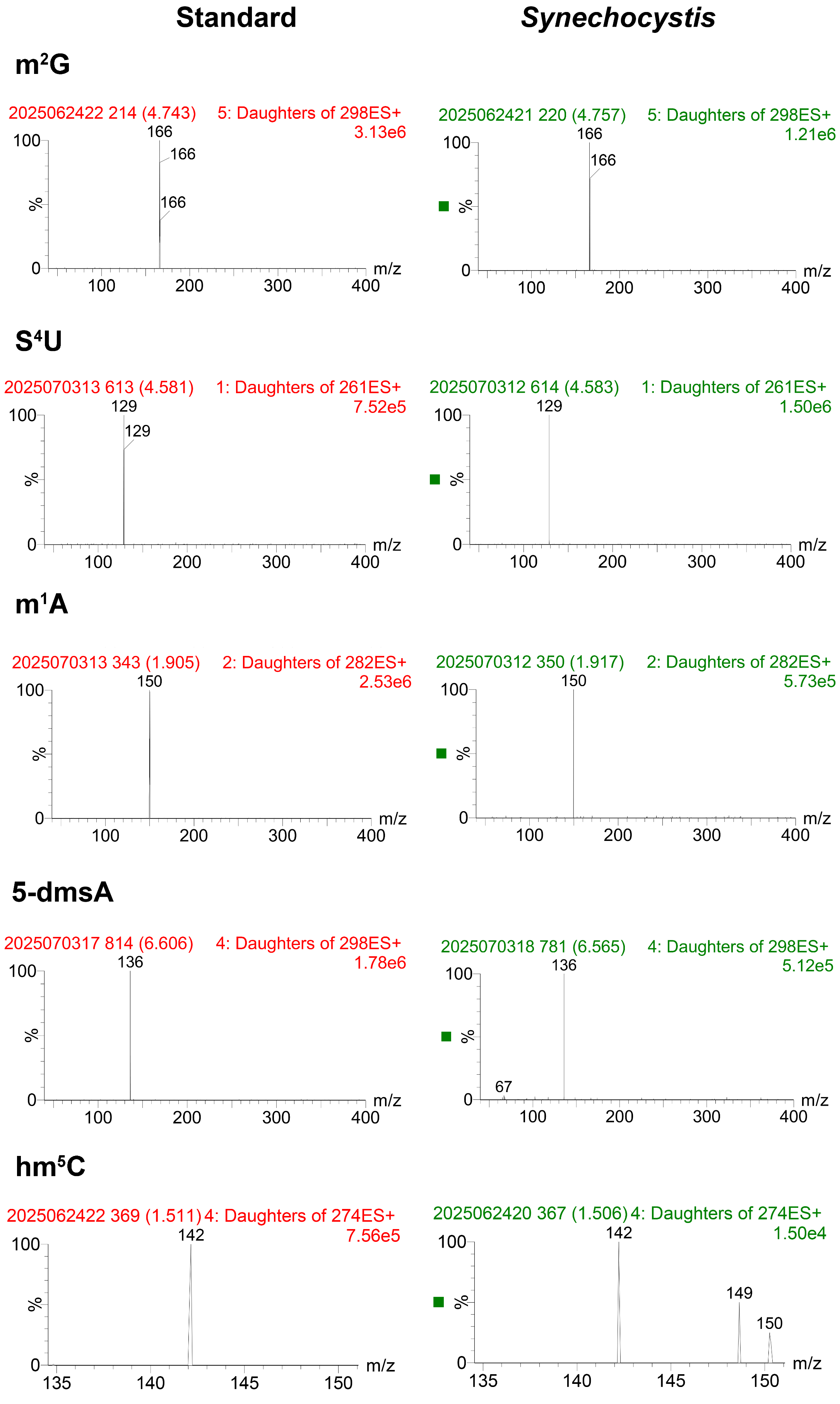


**Figure S2**. Veriﬁcation of nucleosides by comparing the tandem MS/MS spectra from the *Synechocystis* RNA samples with those of synthesized nucleoside standards. X-axis: retention time; Y-axis: percentage of base peak intensity (%).


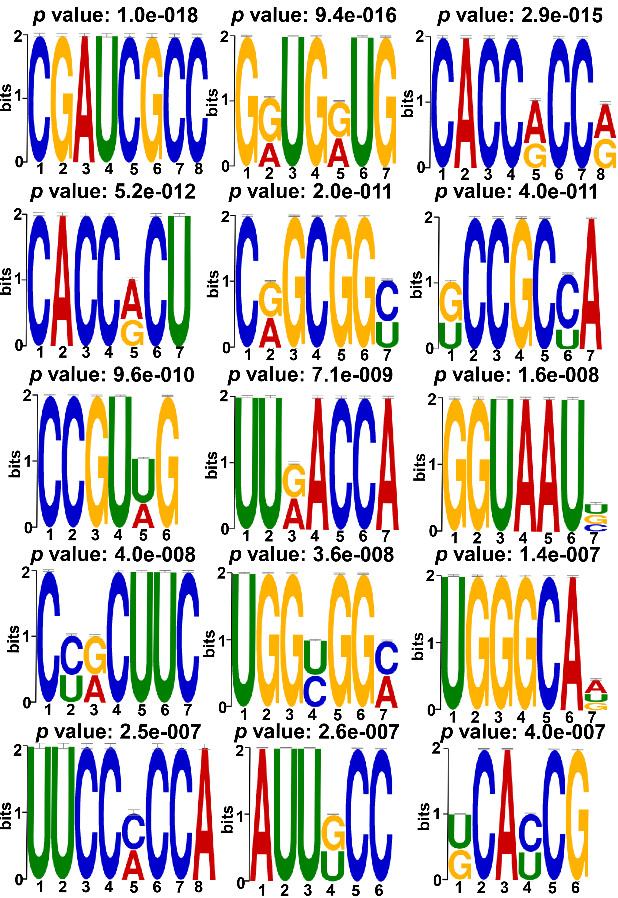

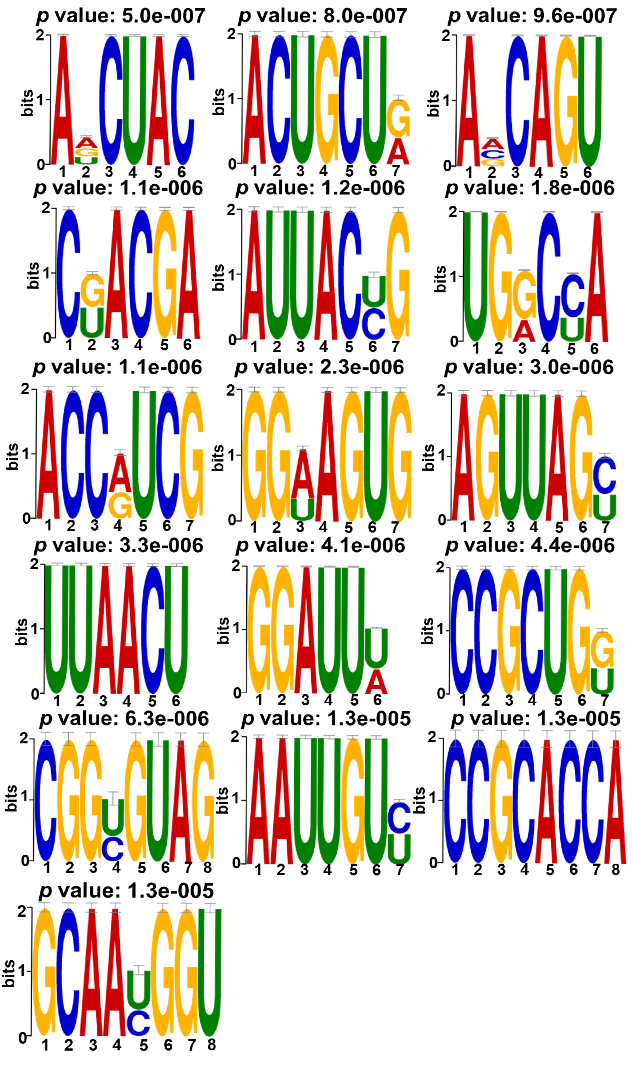


**Figure S3.** Sequence logo representations of the enriched consensus motifs identified from m^5^C peaks. Motifs and associated *p* values were generated using DREME software.


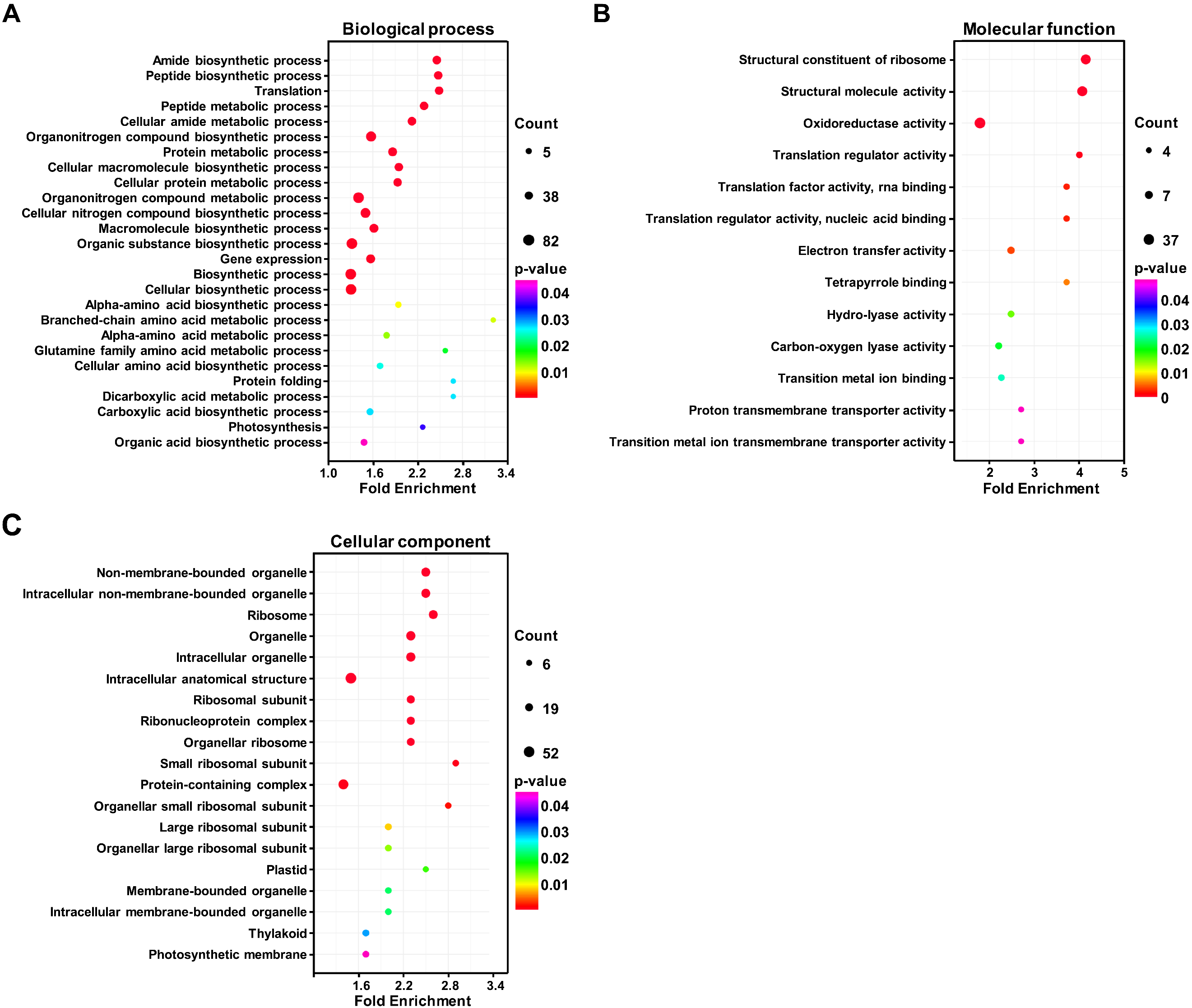


**Figure S4.** GO enrichment analysis of m^5^C genes in *Synechocystis* according to biological process (A), molecular function (B), cellular component (C).


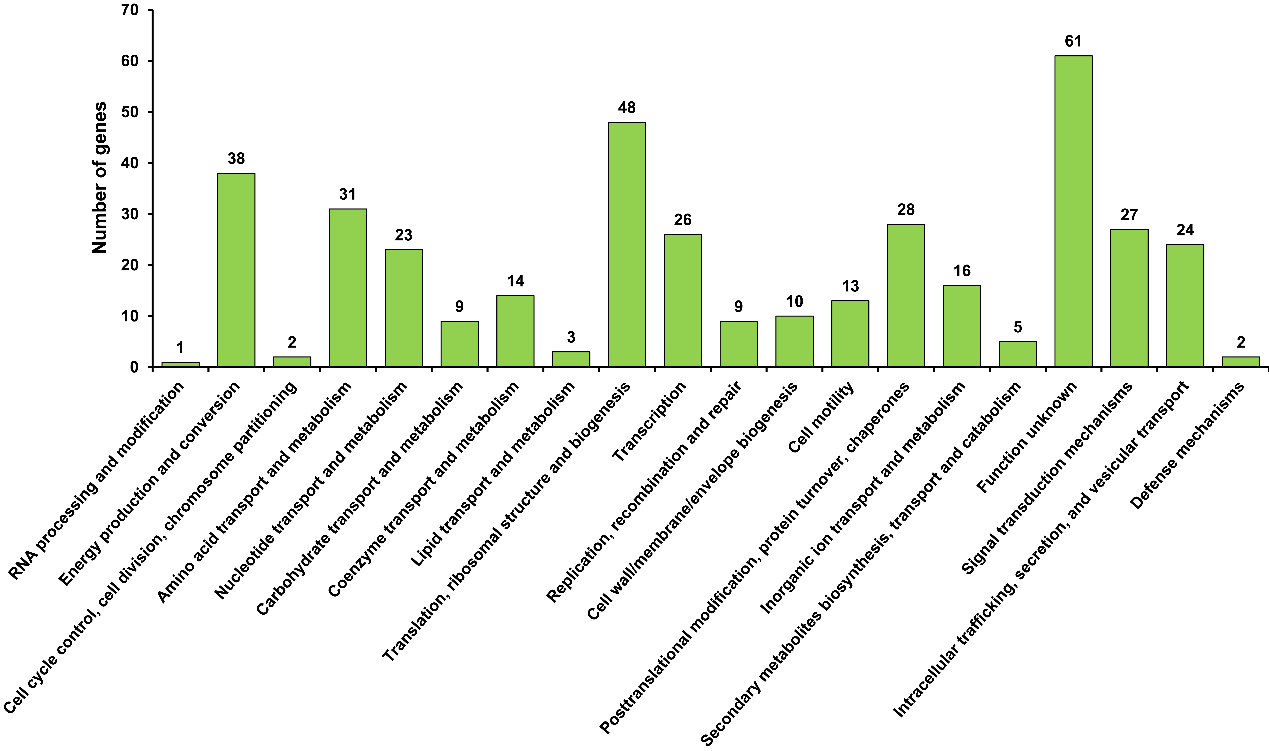


**Figure S5.** COG annotation of m^5^C genes in *Synechocystis*.


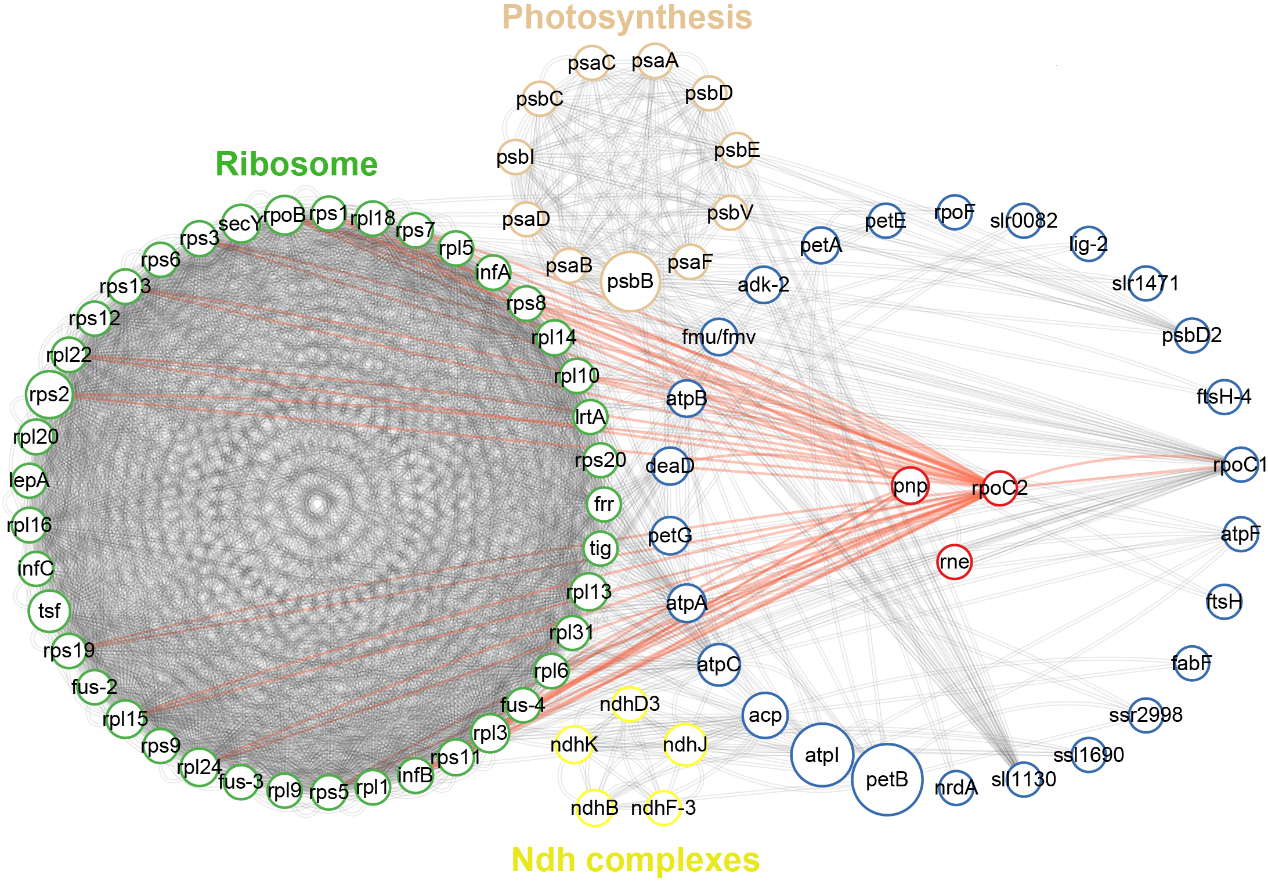


**Figure S6.** Interaction network of m^5^C genes in *Synechocystis* associated with ribosome, Ndh complexes and photosynthesis.

**
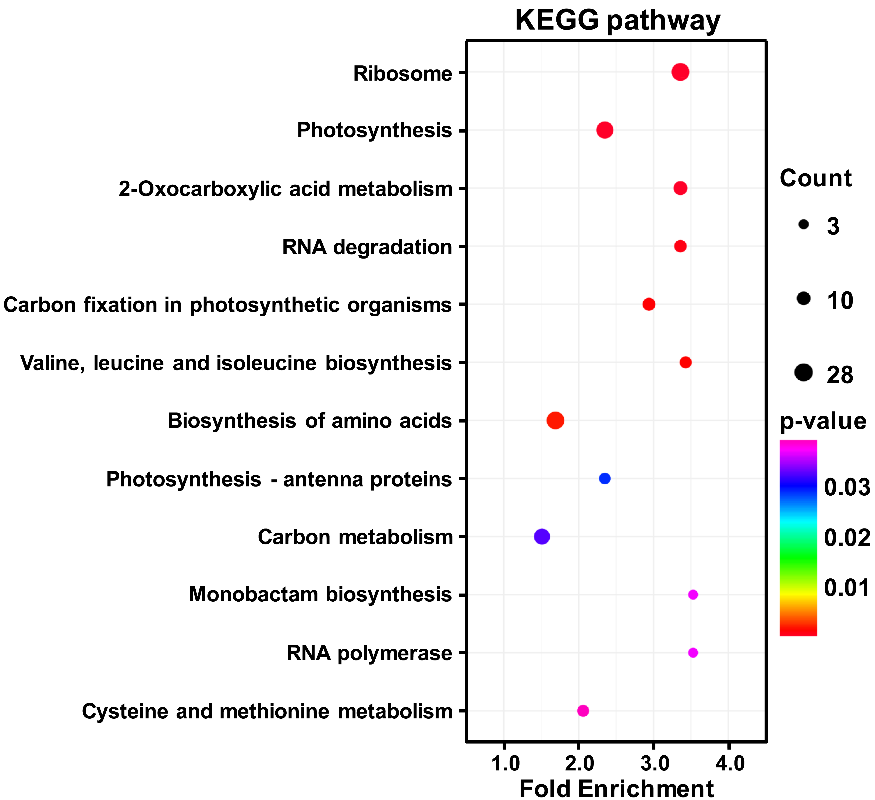
**

**Figure S7.** KEGG pathway enrichment analysis of m^5^C genes in *Synechocystis*.


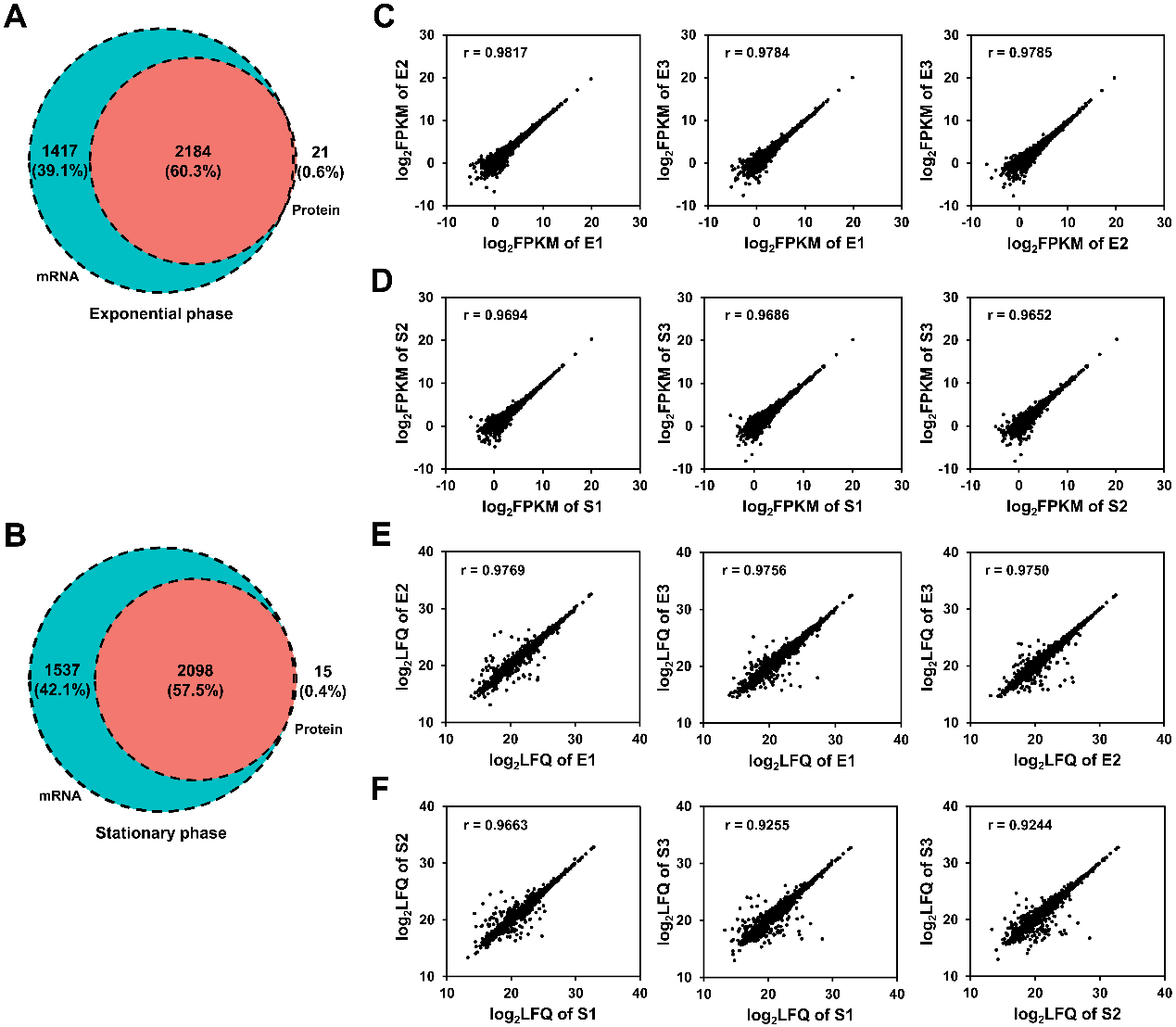


**Figure S8.** Comparison of transcriptomic and proteomic datasets. (A-B) Venn diagrams illustrating the overlap of genes identified at the transcriptomic and proteomic levels in the exponential (A) and stationary (B) phases. (C-D) Pairwise correlation of mRNA abundance among three biological replicates in the exponential (C) and stationary (D) phases. (E-F) Pairwise correlation of protein abundance among three biological replicates in the exponential (E) and stationary (F) phases. Correlation coefficients were calculated using spearman’s correlation coeﬃcient test, based on log_2_-transformed FPKM values (transcriptomics) and LFQ intensities (proteomics).
